# A Scalable BaTiO_3_ Nanocoating Strategy for Cost‐Effective and Stable Sulfide‐Based All‐Solid‐State Batteries

**DOI:** 10.1002/advs.74722

**Published:** 2026-03-04

**Authors:** Wenjin Li, Qingmei Xiao, Shiming Huang, Ruonan Zhang, Hong Yu, Donghao Liang, Kaiyuan Deng, Cheng Liu, Beisen Chen, Puxi An, Guangliang Gary Liu

**Affiliations:** ^1^ Guangdong Provincial Key Laboratory of New Energy Materials Service Safety, College of Materials Science and Engineering Shenzhen University Shenzhen China

**Keywords:** air stability, all‐solid‐state batteries, BaTiO_3_ nanocoating, fast‐charging capability, sulfide electrolytes

## Abstract

Sulfide‐based all‐solid‐state batteries (ASSBs) are promising for next‐generation energy storage due to their high energy density and intrinsic safety, yet their practical deployment is limited by the high cost of sulfide electrolytes, poor air stability, and interfacial degradation with nickel‐rich cathodes. Here, a scalable and cost‐effective BaTiO_3_ (BTO) nanocoating strategy for Li_5.5_PS_4.5_Cl_1.5_ (LPSC1.5) electrolytes is presented, achieved through a rapid 10 min ball‐milling process. The uniform ∼100 nm BTO layer reduces electrolyte cost by approximately 8.1% while maintaining high ionic conductivity (8.81 mS cm^−1^). The coating significantly enhances air stability by suppressing H_2_S evolution and preserving conductivity after exposure. Combined experimental and finite element analyses reveal that the BTO layer homogenizes charge distribution, inhibits space‐charge layer formation, and mitigates interfacial side reactions, leading to improved electrochemical and mechanical robustness. When paired with PCNCM83 cathodes, the modified electrolytes enable ASSBs with exceptional rate capability and ultralong cycling stability exceeding 10 000 cycles at 7 C. This universal nanocoating approach is compatible with various sulfide electrolytes and cathode chemistries, offering a viable pathway toward the scalable commercialization of high‐performance solid‐state batteries.

## Graphical abstract

A rapid and scalable 10 min BaTiO_3_ (BTO) nanocoating uniformly encapsulates Li‐P‐S‐X sulfide electrolytes, serving as a dielectric “shield” that enhances air stability, reduces cost, and mitigates interfacial degradation. This multifunctional coating enables fast‐charging, durable all‐solid‐state batteries with ultralong cycling stability (>10 000 cycles at 7 C) and broad compatibility across diverse sulfide electrolytes and Ni‐rich cathodes—offering a nanoscience‐driven, industrially viable pathway toward scalable solid‐state battery manufacturing.

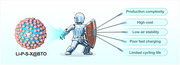

## 1 Introduction

All‐solid‐state batteries (ASSBs) are widely recognized as one of the most promising next‐generation energy storage technologies, combining high energy density, intrinsic safety, and design flexibility beyond conventional liquid‐electrolyte lithium‐ion batteries [1, 2, 3]. Among the various solid electrolytes, sulfide‐based systems such as argyrodite‐type Li‐P‐S‐X (X = Cl, Br) have attracted particular attention due to their high ionic conductivity (>5 mS cm^−1^), excellent mechanical deformability, and compatibility with low‐temperature processing [4, 5]. These advantages render sulfide solid electrolytes (SSEs) well‐suited for integration with high‐voltage, Ni‐rich layered oxide cathodes [6, 7]. Nevertheless, the large‐scale commercialization of sulfide‐based ASSBs remains constrained by persistent challenges including high material cost, poor air stability, and severe interfacial degradation during cycling [8, 9, 10, 11].

The high cost of key precursor materials such as lithium sulfide (Li_2_S) drives SSEs prices to nearly 291 USD per kg—substantially exceeding those of commercial liquid electrolytes [12, 13, 14]. Moreover, SSEs are highly sensitive to atmospheric moisture, undergoing rapid hydrolysis that generates toxic hydrogen sulfide (H_2_S) gas and induces chemical decomposition at the surface [15, 16, 17, 18]. This instability complicates fabrication, storage, and handling, thereby inflating manufacturing costs and raising safety risks. Additionally, solid‐solid interfacial contact between the SE and active materials often deteriorates over cycling. This problem is particularly pronounced with high‐nickel cathodes (e.g., LiNi_0.8_Co_0.1_Mn_0.1_O_2_ (NCM811), LiNi_0.83_Co_0.11_Mn_0.06_O_2_ (NCM83)), where interfacial reactions and space‐charge layer (SCL) formation produce resistive interphases that hinder Li^+^ transport [19, 20, 21, 22, 23]. Together, these issues severely degrade mechanical and electrochemical integrity, compromising fast‐charging capability and long‐term stability—key barriers to practical ASSB deployment.

To mitigate these limitations, several modification strategies have been explored, including heteroatom substitution and surface coatings [24, 25, 26, 27]. While oxide or halide doping can partially enhance air stability and reduce cost, these approaches frequently lower ionic conductivity or introduce new interfacial resistance [28, 29]. Chemical coatings using agents such as LiPO_2_F_2_ or LiC_2_O_4_BF_2_ have shown potential to alleviate interfacial reactions, yet they often require complex synthesis or lead to conductivity loss [30, 31]. Moreover, existing modification strategies primarily target interfacial resistance reduction through particle migration‐dominated transport models, neglecting the intrinsic regulation of energy states at the interface. Recent theoretical advances have demonstrated that energy transmission in confined systems is governed by state accessibility and resonance rather than mere particle migration, highlighting the need for interfacial engineering strategies tailored to energy state optimization. [32] Therefore, developing a simple, scalable, and cost‐effective approach that simultaneously preserves high ionic conductivity, enhances air stability, and stabilizes electrode‐electrolyte interfaces remains a pressing imperative.

Here, we report a universal BaTiO_3_ (BTO) nanocoating strategy that effectively resolves these challenges in a single, scalable step. Using a rapid 10 min ball‐milling process, a conformal ∼100 nm dielectric BTO layer is uniformly applied to Li_5.5_PS_4.5_Cl_15_ (LPSC1.5) particles, yielding LPSC1.5@10BTO. This nanocoating reduces electrolyte cost by 8%–12% while preserving high ionic conductivity (>7 mS cm^−1^). Acting as a moisture barrier, it enhances air stability by suppressing H_2_S emission by 67%. Finite element simulations and interfacial analyses confirm that the dielectric nature of BTO homogenizes charge distribution, suppresses SCL formation, and mitigates side reactions—leading to improved interfacial and mechanical stability. Consequently, cells combining polycrystalline NCM83 (PCNCM83) cathodes with LPSC1.5@10BTO electrolytes achieve exceptional performance, including fast‐charging operation up to 10 C and ultralong cycling stability exceeding 10 000 cycles.

Beyond this specific system, the BTO nanocoating demonstrates broad applicability across diverse sulfide electrolytes—such as commercial Li_6_PS_5_Cl (LPSC) and Li_5.5_PS_4.5_Cl_0.8_Br_0.7_ (LPSCB)—and Ni‐rich cathodes, including single‐crystal NCM811 (SCNCM811) and NCM83 (SCNCM83). The simplicity, cost efficiency, and universal compatibility of this strategy establish it as a practical platform for scalable manufacturing of high‐performance sulfide‐based ASSBs.

## 2 Results and Discussions

### 2.1 Cost, Structure, Morphology and Li^+^ Conductivity

**FIGURE 1:**
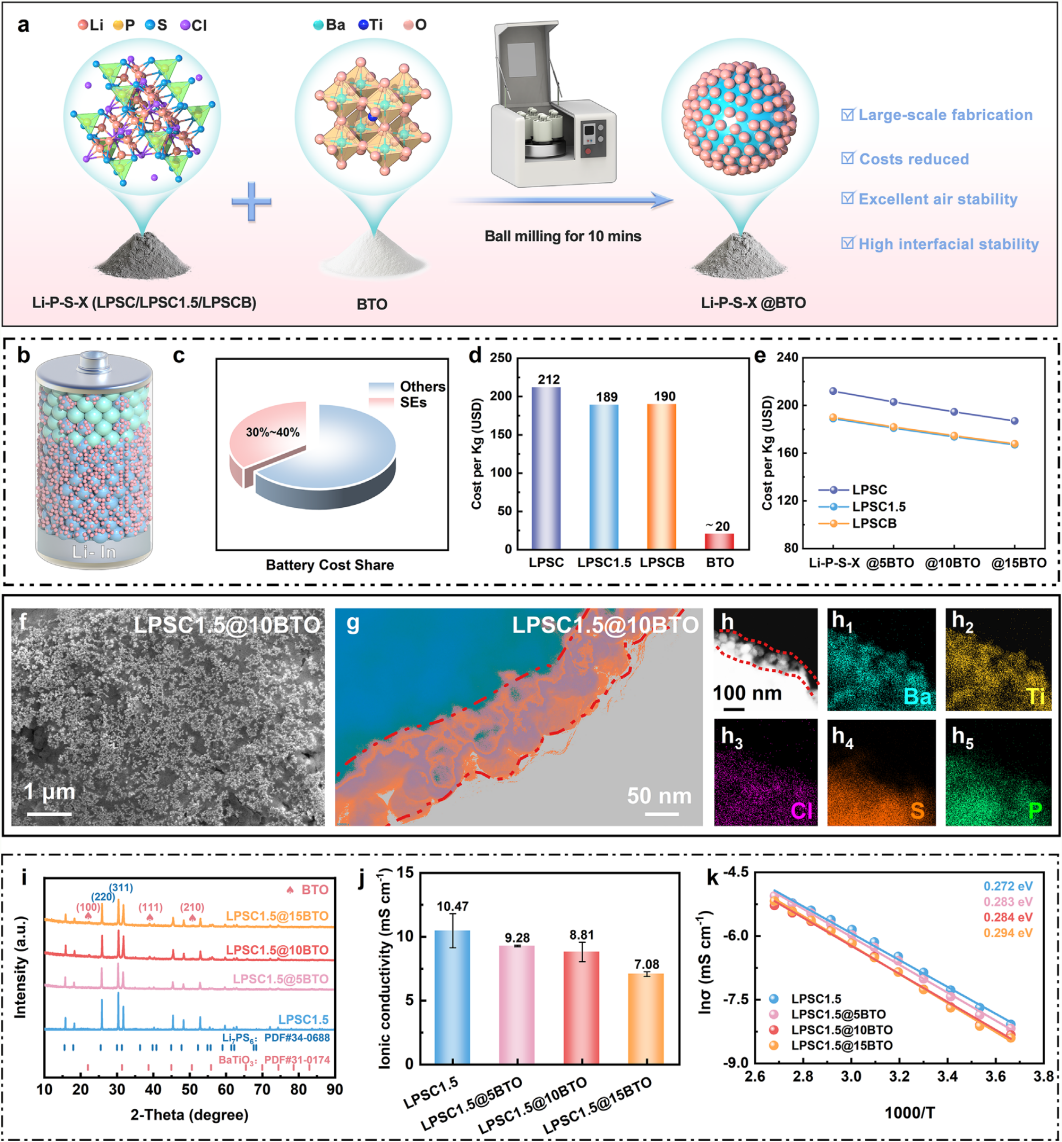
Materials preparation. (a) Schematic illustration of the efficient nano‐BTO coating strategy for SSEs and its multifunctional roles in ASSBs. (b) Schematic illustration of the structure of ASSBs. (c) The cost proportion of SEs. (d) The raw material costs of Li‐P‐S‐X SSEs and BTO. (d) The cost of coated Li‐P‐S‐X electrolytes with varying amounts of BTO. The (f) SEM image and (g,h) TEM images of LPSC1.5@10BTO with corresponding EDS elemental mapping of (h_1_‐h_5_) Ba, Ti, Cl, S, and P, respectively. (i) XRD patterns of LPSC1.5@xBTO (x = 0, 5, 10, and 15). (j) Ion conductivity of LPSC1.5@xBTO (x = 0, 5, 10, and 15) at 25°C (bar chart). (k) Arrhenius plots of ionic conductivity for LPSC1.5@xBTO (x = 0, 5, 10, and 15).

Figure 1a schematically illustrates the multifunctional BTO nanocoating strategy for Li‐P‐S‐X SSEs. The coating is applied via a simple 10 min ball‐milling process. Because electrolyte materials account for 30%–40% of the total cell cost (Figure 1b,c), reducing electrolyte expense is essential for the commercialization of ASSBs. BTO serves as an ideal cost‐efficient coating material, available commercially at approximately 20 USD kg^−1^—an order of magnitude lower than Li‐P‐S‐X SSEs (189‐212 USD kg^−1^; Figure 1d and Tables S1 and S2) [12, 14, 28]. Cost modeling (Figure 1e) shows that increasing BTO content from 0 to 15 wt.% progressively reduces the total electrolyte cost from 189 to 167 USD kg^−1^, corresponding to an 11.6% reduction. This tunable and scalable approach thus enables precise cost optimization while maintaining electrochemical functionality.

Lab‐synthesized LPSC1.5 was selected as a representative sulfide electrolyte due to its high ionic conductivity approaching 10 mS cm^−1^. The morphological and microstructural evolution of LPSC1.5 following BTO modification was characterized by scanning electron microscopy (SEM) and high‐resolution transmission electron microscopy (HRTEM). SEM images of commercial BTO nanoparticles (Figure S1) reveal nearly spherical particles with an average diameter of ∼50 nm. As shown in Figure 1f and Figure S2, the BTO nanoparticles are homogeneously distributed on the LPSC1.5 surface at loadings of 5, 10, and 15 wt.%. Energy‐dispersive X‐ray spectroscopy (EDS) mapping confirms uniform elemental distributions of Ti, Ba, and O across the composite particles. HRTEM images (Figure 1g; Figure S3) further reveal a continuous ∼100 nm BTO coating layer conformally encapsulating the sulfide particles. This nanoscale uniformity is essential for minimizing interfacial resistance and improving air stability, as discussed in later sections. Elemental mapping (Figure 1h) corroborates intimate interfacial contact between BTO and LPSC1.5 through co‐localized Ba, Ti, Cl, S, and P signals, confirming that BTO is strictly confined to the LPSC1.5 surface without bulk penetration. The presence of BTO is further confirmed by the Ti 2p, Ba 3d, and O 1s peaks in X‐ray photoelectron spectroscopy (XPS) spectra (Figures S4 and S5). X‐ray diffraction (XRD, Figure 1i) patterns of LPSC1.5@xBTO (x = 0–15) exhibit distinct peaks corresponding to both LPSC1.5 [(220), (311)] [33] and BTO [(100), (111), (210)] [34] without secondary phases. These results collectively confirm the absence of chemical or electrochemical interactions between BTO and LPSC1.5 during the ball‐milling process and further verify that the coating procedure preserves the intrinsic crystalline structure of LPSC1.5 as well as its Li^+^ conduction pathways.

To evaluate the influence of the coating on ionic transport, electrochemical impedance spectroscopy (EIS) was performed at room temperature (Figure 1j). LPSC1.5@10BTO exhibits a high ionic conductivity of 8.81 mS cm^−1^, closely matching that of pristine LPSC1.5 (10.47 mS cm^−1^). Even at 15 wt.% BTO loading, conductivity remains 7.08 mS cm^−1^, sufficient for practical high‐rate operation. Arrhenius analysis (Figure 1k) across 0–100°C (Figures S6 and S7) yields activation energies of 0.283, 0.284, and 0.294 eV for 5, 10, and 15 wt.% BTO loadings, respectively, comparable to 0.272 eV for pristine LPSC1.5. The negligible increase in activation energy indicates that the BTO coating does not impede Li^+^ migration, likely due to enhanced interfacial ionic conduction facilitated by the nanoscale dielectric layer [35]. In contrast to Li^+^‐conductive coatings (e.g., LiPO_2_F_2_ and LiC_2_O_4_BF_2_), which lead to substantial reductions in electrolyte ionic conductivity, our BTO nanocoating preserves high ionic conductivity while simultaneously enhancing physical stability—thereby underscoring the rationality and effectiveness of this design strategy.

Overall, the BTO nanocoating achieves a synergistic balance between cost efficiency, structural compatibility, and high Li^+^ conductivity. This design not only mitigates the economic and interfacial challenges of SSEs but also establishes a foundation for subsequent investigations into air stability, interfacial chemistry, and long‐term cycling durability.

### 2.2 Moisture Stability of BTO‐Coated Electrolytes

The poor air and moisture stability of SSEs remains a major challenge, as these materials readily react with atmospheric moisture to generate toxic H_2_S gas and insulating byproducts, leading to structural degradation and severe loss of electrochemical performance [36]. To systematically evaluate the protective function of the BTO nanocoating, we performed comprehensive morphological, structural, chemical, and electrochemical characterizations of LPSC1.5@10BTO, demonstrating its remarkable resistance to moisture‐induced degradation.

**FIGURE 2:**
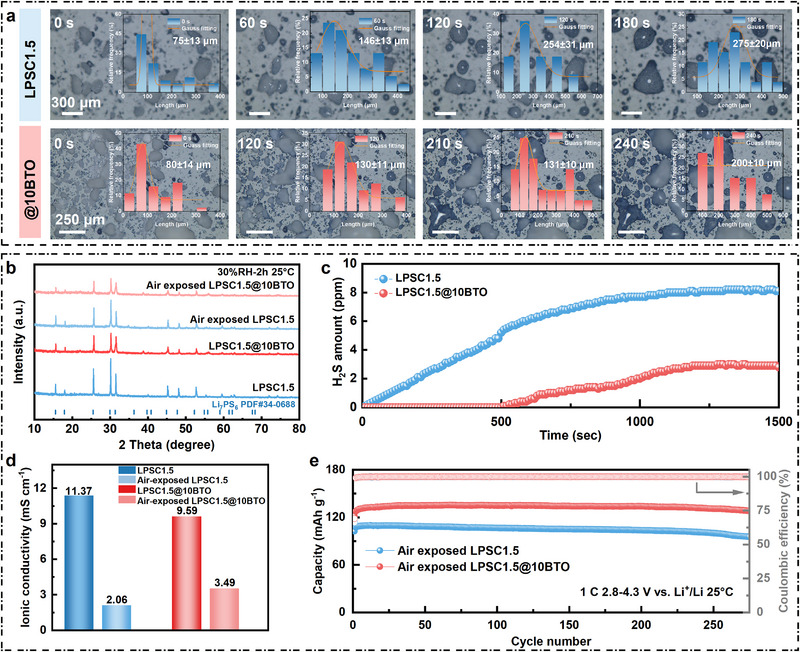
Verifying the tolerance against moist air. (a,b) The macroscopic morphology of LPSC1.5 and LPSC1.5@10BTO powder changes with time in air environment (25°C, 70 ± 5% RH). (c) The quantity of H_2_S generated from LPSC1.5 and LPSC1.5@10BTO pellets (pelletizing pressures is 300 Mpa) when exposed in the wet air (25°C, 30 ± 5% RH). (d) Ionic conductivity of LPSC1.5 and LPSC1.5@10BTO after being exposed in the wet air (25°C, 30 ± 5% RH) for 2 h. (e) Cycling performance curves of ASSBs based on exposed LPSC1.5 and LPSC1.5@10BTO electrolyte.

The morphological evolution of pristine and BTO‐coated LPSC1.5 during air exposure (25°C, 70 ± 5% RH) was monitored in real time (Figure 2a). Pristine LPSC1.5 exhibited rapid particle agglomeration and growth, with the average particle size increasing from 75 ± 13 µm (0 s) to 275 ± 20 µm (180 s), accompanied by severe coalescence attributed to moisture‐driven reactions (Video S1). In contrast, LPSC1.5@10BTO showed markedly slower agglomeration, with the particle length increasing only from 80 ± 14 µm (0 s) to 200 ± 10 µm after 240 s (Video S2), confirming that the BTO layer effectively blocks moisture penetration.

X‐ray diffraction (Figure 2b) further confirmed the structural stability of the coated electrolyte. After air exposure (30 ± 5% RH, 2 h, 25°C), pristine LPSC1.5 displayed pronounced peak broadening and new impurity peaks, indicating phase decomposition. In contrast, both as‐prepared and air‐exposed LPSC1.5@10BTO retained the characteristic reflections of LPSC1.5 and BTO without detectable impurities, demonstrating that the nanocoating preserves the crystalline integrity of the sulfide phase under humid conditions.

The release of H_2_S during air exposure was quantitatively assessed (Figure 2c). Pristine LPSC1.5 rapidly released H_2_S, reaching ∼8 ppm within 1500 s, while LPSC1.5@10BTO showed a dramatically reduced emission of ∼3 ppm—a 63% suppression. This substantial reduction highlights the coating's effectiveness in preventing moisture‐sulfide reactions and improving environmental safety.

EIS in Figure 2d and Figure S8 revealed that the ionic conductivity of pristine LPSC1.5 decreased sharply from 11.37 to 2.06 mS cm^−1^ after air exposure. In contrast, LPSC1.5@10BTO retained 3.49 mS cm^−1^ from an initial 9.59 mS cm^−1^, maintaining 18.3% higher conductivity retention than the pristine sample after exposure. These results demonstrate that the BTO nanolayer preserves Li^+^ conduction pathways by preventing surface degradation and maintaining structural continuity under humid conditions.

Visual and chemical analyses support these findings. After 2 h exposure (30 ± 5% RH), pristine LPSC1.5 pellets showed evident surface deterioration (Figure S9a), while LPSC1.5@10BTO retained structural integrity (Figure S9b). XPS spectra (Figure S10) reveal significant oxidation of sulfur and phosphorus species in pristine samples, whereas the coated electrolyte maintained the sulfide signatures and exhibited characteristic Ti‐O and Ba‐O features, confirming chemical stability [37].

To correlate these effects with practical performance, PCNCM83/LPSC1.5@xBTO/Li‐In full cells were assembled using air‐exposed electrolytes (1 C, 2.8–4.3 V vs. Li^+^/Li, 25°C, Figure 2e). Cells with pristine LPSC1.5 suffered rapid capacity decay (100.2 mAh g^−1^ after 250 cycles, Coulombic efficiency (CE) < 99.9%), while those using LPSC1.5@10BTO maintained 130.9 mAh g^−1^ with a CE above 99.9% over 250 cycles. This superior performance confirms that the BTO coating enables SSEs to retain electrochemical integrity even after ambient exposure—an essential advantage for scalable processing and long‐term storage [38]. Collectively, the BTO nanocoating imparts exceptional moisture stability to LPSC1.5 by mitigating particle agglomeration, preserving crystallinity, suppressing H_2_S evolution, maintaining high Li^+^ conductivity, and ensuring durable electrochemical performance.

### 2.3 Fast‐Charging Performance and Long‐Term Cycling Stability

To elucidate the influence of BTO modification on rate capability and cycling durability, we systematically investigated the electrochemical performance of LPSC1.5‐based all‐solid‐state cells. Lab‐synthesized PCNCM83, with an average particle size (D_50_) of 3–5 µm (Figure S11), was selected as the cathode due to its excellent interfacial compatibility with SSEs and suitability for high‐rate operation [39].

**FIGURE 3:**
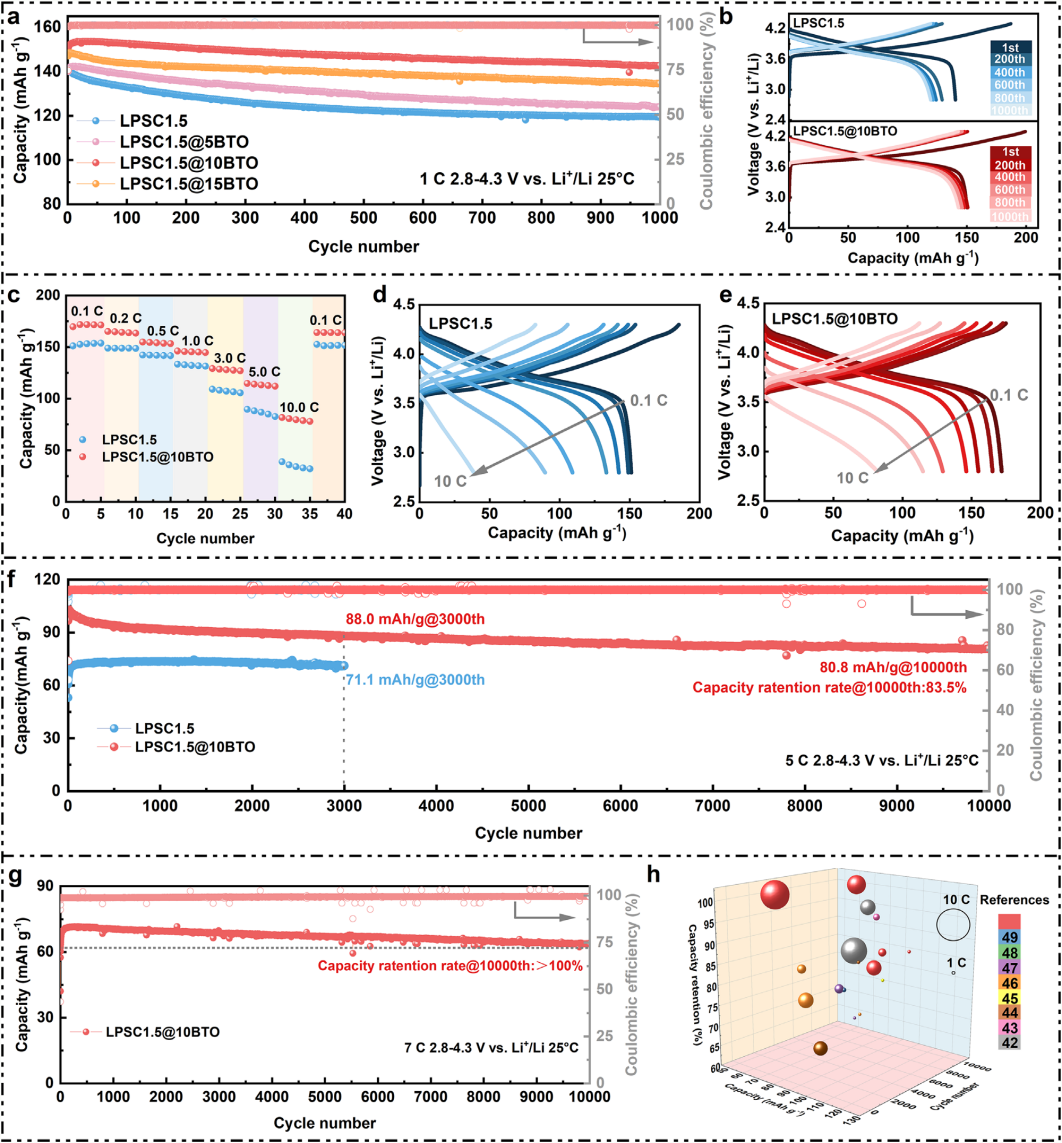
PCNCM83 ASSB cells employing LPSC1.5@xBTO. (a) Cycle performance of PCNCM83/LPSC1.5@xBTO (x = 0, 5, 10, and 15)/LiIn at 1 C within the voltage range of 2.8–4.3 V vs. Li^+^/Li. (b) Charge‐discharge profiles of PCNCM83/LPSC1.5@xBTO (x = 0 and 10)/LiIn. (c) Rate performance and (d,e) charge–discharge profiles of PCNCM83/LPSC1.5@xBTO (x = 0 and 10)/LiIn. (f) Cycling performance of PCNCM83/LPSC1.5@xBTO (x = 0 and 10)/LiIn at 5 C. (g) Cycling performance of PCNCM83/LPSC1.5@10BTO/LiIn at 7 C. (h) Comparison of the fast‐charging performance and cycling stability of PCNCM83/LPSC1.5@10BTO/LiIn cells with previously reported values in the literature [42, 43, 44, 45, 46, 47, 48, 49]. The red color represents the performance of this work.

As shown in Figure 3a, the PCNCM83/LPSC1.5@xBTO/LiIn cells (x = 5, 10, 15 wt.%) deliver initial discharge capacities of 140.0, 150.0, and 147.1 mAh g^−1^ at 1 C (2.8‐4.3 V vs. Li^+^/Li, 25°C), with initial Coulombic efficiencies (ICE) of 75.24%, 75.30%, and 75.22%, respectively, and capacity retentions of 88.6%, 94.9%, and 91.6% after 1000 cycles. In contrast, cells with pristine LPSC1.5 exhibit a comparable initial discharge capacity (140.3 mAh g^−1^) but a lower ICE (75.03%) and only 85.1% capacity retention after 1000 cycles. The superior ICE and cycling stability of the LPSC1.5@10BTO‐based cell underscore the optimized 10 wt.% BTO coating, which achieves an ideal balance between ionic conductivity, interfacial compatibility, and cost efficiency.

Galvanostatic charge‐discharge (GCD) profiles (Figure 3b) show that pristine LPSC1.5 suffers from pronounced voltage hysteresis and capacity fading upon cycling, while LPSC1.5@10BTO maintains narrow voltage plateaus and stable capacity even after hundreds of cycles. Rate tests (Figure 3c) further highlight the remarkable kinetic performance: LPSC1.5@10BTO achieves 169.7 mAh g^−1^ at 0.1 C—18.6 mAh g^−1^ higher than pristine LPSC1.5—and retains 114.8 mAh g^−1^ at 5 C and 81.7 mAh g^−1^ at 10 C. Quantitative comparisons with previously reported sulfide‐based ASSBs (Table S3) demonstrate that the present system exhibits one of the highest rate capabilities reported to date among argyrodite‐based ASSBs. The corresponding GCD curves (Figure 3d,e) confirm minimal polarization even under extreme current densities, indicative of accelerated Li^+^ transport and reduced interfacial resistance [40]. Furthermore, the Galvanostatic intermittent titration technique (GITT) measurements (Figure S12) reveal that the average Li^+^ diffusion coefficient (D_Li_^+^) of LPSC1.5@10BTO‐based cell reaches 1.16 × 10^−9^ cm^2^ s^−1^, representing a 1.34‐fold enhancement compared to that of pristine LPSC1.5‐based cell (8.64 × 10^−9^ cm^2^ s^−1^), thereby confirming that the BTO nanocoating boosts interfacial Li^+^ transport efficiency during charge‐discharge cycling.

The fast‐charging capability of LPSC1.5@10BTO is particularly striking. At 3 C, the cell retains 103.3 mAh g^−1^ after 5000 cycles (85.1% retention, Figure S13), and at 5 C, it delivers 80.8 mAh g^−1^ after 10 000 cycles (83.5% retention, Figure 3f). By comparison, pristine LPSC1.5 rapidly degrades to 92.3 mAh g^−1^ after 5000 cycles at 3 C and 71.1 mAh g^−1^ after only 3000 cycles. Under even harsher conditions—7 C (Figure 3g) and 10 C (Figure S14)—LPSC1.5@10BTO remarkably maintains >100% capacity retention after 10 000 cycles, a phenomenon attributed to interfacial stabilization and gradual electrochemical activation [41]. The nearly unchanged GCD voltage profiles over extended cycling further demonstrate its outstanding interfacial resilience. Elevated temperature cycling at 50°C (Figure S15) reinforces these findings: LPSC1.5@10BTO retains 89.4% of its capacity after 500 cycles, outperforming pristine LPSC1.5 (77.4%).

A three‐dimensional rate‐cycling performance map (Figure 3h) vividly summarizes the comprehensive electrochemical advantages of the BTO‐coated electrolyte. Its combined capacity retention and rate tolerance exceed the performance reported for state‐of‐the‐art sulfide‐based NCM systems (Table S4) [42, 43, 44, 45, 46, 47, 48, 49]. This exceptional behavior originates from the dual function of the BTO nanocoating: 1) interfacial stabilization through dielectric screening and chemical passivation, and 2) facilitated Li^+^ migration via maintained ionic pathways at the coating‐electrolyte boundary.

### 2.4 Interfacial Structure and Mechanistic Insights

The electrochemical performance of ASSBs is intrinsically governed by the structural integrity and interfacial compatibility between SSEs and electrodes, and SSEs’ intrinsic oxidation resistance. To reveal the superior behavior of LPSC1.5@10BTO, we first assessed the oxidative stability of the electrolytes using cyclic voltammetry (CV) (Figure S16). The integrated oxidation peak area of LPSC1.5@10BTO is markedly smaller than that of pristine LPSC1.5 over the first three cycles, indicating effective suppression of oxidative decomposition in the SSEs by the BTO modification. This enhanced oxidative stability not only improves the long‐term chemical and electrochemical robustness of the electrolyte but also contributes to maintaining cathode structural integrity and stabilizing the cathode‐electrolyte interfacial chemistry. We then used advanced characterization and simulation techniques to analyze its interfacial charge dynamics and structural evolution.

**FIGURE 4:**
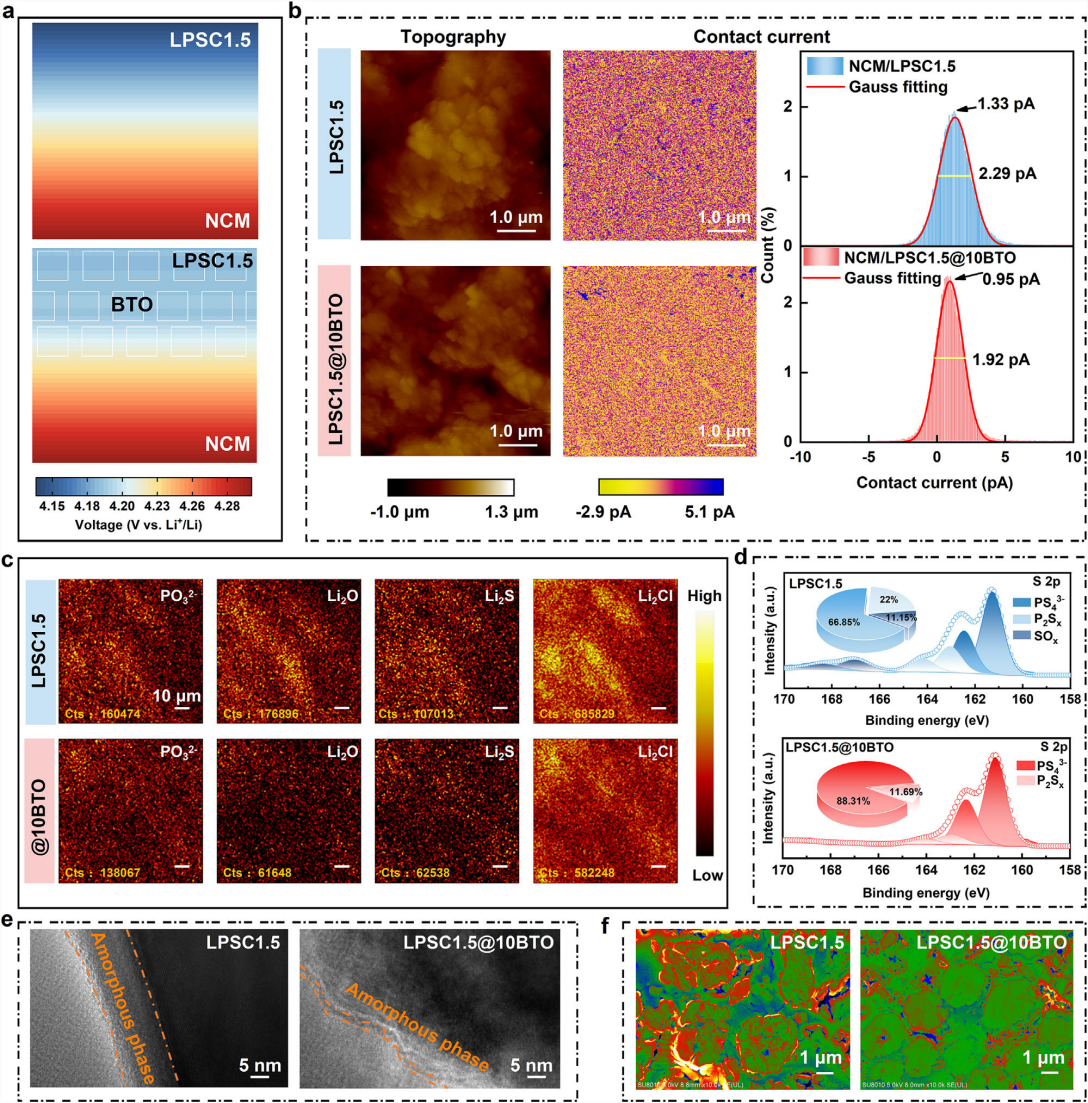
Interfacial electro‐chemo‐mechanics evolution in the PCNCM83/ LPSC1.5@xBTO ASSB cells during cycling. (a) Simulation results of the internal electrical field for the PCNCM83/LPSC1.5 and PCNCM83/LPSC1.5@BTO interfaces. (b) Surface topography, and I_c_ images of the pristine interface of PCNCM83/LPSC1.5 and PCNCM83/LPSC1.5@BTO. (c) TOF‐SIMS chemical species images and (d) S 2p XPS spectra from PCNCM83/LPSC1.5 and PCNCM83/LPSC1.5@BTO composite after 1000 cycles at 1 C. (e) HRTEM images of PCNCM83 in PCNCM83/LPSC1.5 and PCNCM83/LPSC1.5@BTO composite after 1000 cycles at 1 C. (f) Cross‐sectional SEM images at the cathode/SE interface after 1000 cycles at 1 C.

Finite element simulations were first conducted on the NCM83/LPSC1.5 system to investigate the effect of the BTO coating on SCL formation under applied bias (Figure 4a; Figure S17). In the pristine configuration, a pronounced SCL develops at the NCM83/LPSC1.5 interface, characterized by a steep potential gradient and highly non‐uniform potential distribution—signatures of hindered Li^+^ transport. In contrast, when BTO is introduced between NCM83 and LPSC1.5, the high‐dielectric layer generates an opposing built‐in electric field that counteracts the external field. This screening effect reduces the interfacial potential gradient, homogenizes the potential distribution, and significantly suppresses SCL formation [50].

To experimentally validate these predictions, in‐situ evolution of EIS spectra to quantitatively assess interfacial reaction kinetics and SCL formation (Figures S18 and S19). Nyquist plots demonstrate that the total cell impedance of the LPSC1.5‐based battery increases markedly during cycling, reaching a maximum of ∼42.61 Ω at full charge (4.3 V vs. Li^+^/Li). In contrast, the LPSC1.5@10BTO cell exhibits significantly suppressed impedance growth, maintaining a total impedance consistently below ∼20.4 Ω across all states of charge. This pronounced stabilization confirms that the BTO dielectric nanolayer effectively mitigates both SCL formation and dynamic interfacial degradation, thereby preserving low and stable interfacial resistance throughout electrochemical cycling.

Distribution of relaxation times (DRT) analysis was performed on the EIS spectra to resolve the individual contributions of distinct interfacial and bulk processes. Each resolved peak corresponds to a characteristic time constant (τ), which reflects the dominant polarization mechanism at a given timescale: τ_1_ (∼10^−5^ s) is assigned to grain‐boundary resistance within the SSE particles; τ_2_ (∼10^−4^ s) and τ_3_ (∼10^−3^ s) are associated with the anodic solid electrolyte interphase (SEI) and cathode‐electrolyte interphase (CEI), respectively, both arising from heterogeneous charge distribution at electrode‐electrolyte interfaces; τ_4_ (∼10^−1^ s) and τ_5_ (∼1 s) correspond to charge‐transfer resistance at the cathode‐electrolyte and anode‐electrolyte interfaces, respectively; and τ_6_ (>1 s) reflects solid‐state Li^+^ diffusion limitations within the composite cathode bulk. [51] Critically, τ_4_ exhibits strong sensitivity to the SCL effect: accumulation of negative space charge at the cathode‐electrolyte interface during charging induces a counteracting electric field that impedes Li^+^ injection, thereby elevating τ_4_ magnitude. For the BTO‐modified cell, the τ_4_ peak intensity progressively diminishes upon charging, indicating effective suppression of the SCL‐induced interfacial barrier. Conversely, in the BTO‐free LPSC1.5 cell, τ_4_ intensifies markedly during charging, direct evidence of exacerbated SCL formation and associated kinetic hindrance. These DRT‐derived mechanistic insights are in quantitative agreement with complementary finite‐element simulations.

Electrochemical atomic force microscopy (EC‐AFM) was used to probe local charge transport across the NCM83/LPSC1.5 and NCM83/LPSC1.5@10BTO interfaces (Figure 4b). Contact current (I_c_), arising from work function differences and charge rearrangement at the probe‐sample interface, serves as a direct indicator of charge transport uniformity [52]. Pristine NCM83/LPSC1.5 displays a heterogeneous current distribution, with a Gaussian‐fitted I_c_ peak at 1.33 pA, reflecting uneven interfacial transport. In contrast, NCM83/LPSC1.5@10BTO exhibits a narrower I_c_ distribution centered at 0.95 pA, signifying more uniform charge transport. This improvement arises from the dual role of the BTO nanolayer in smoothing the interfacial morphology and enhancing electronic continuity across the interface. Collectively, the results confirm that BTO acts as an effective “buffer layer” that suppresses SCL formation, promotes uniform potential and Li^+^ flux, and mitigates interfacial polarization—key factors driving the exceptional fast‐charging and long‐cycle stability of LPSC1.5@10BTO‐based cells.

Time‐of‐flight secondary ion mass spectrometry (TOF‐SIMS) mapping provided direct evidence of interfacial chemical stabilization (Figure 4c). After prolonged cycling, pristine LPSC1.5 shows strong enrichment of decomposition products such as PO_3_^2−^, Li_2_O, Li_2_S, and Li_4_Cl at the cathode‐electrolyte interface, indicating extensive side reactions and sulfide degradation [47, 53]. In contrast, LPSC1.5@10BTO exhibits a much more uniform elemental distribution without localized byproduct accumulation, confirming that the coating suppresses parasitic interfacial reactions and maintains the chemical integrity of the SSE during cycling. X‐ray photoelectron spectroscopy (XPS, Figure 4d) further supports this conclusion: the oxidized sulfur fraction (P_2_S_x_ and SO_x_) decreases from 33.15% in pristine LPSC1.5 to 11.69% in LPSC1.5@10BTO, with most sulfur remaining in the S^2−^ state (88.31%). Parallel P 2p spectra (Figure S20) corroborates the suppression of oxidative decomposition [54, 55]. This suppression of interfacial degradation originates from BTO's dielectric screening effect, which homogenizes the local electric field distribution across the cathode‐electrolyte interface and thereby attenuates the thermodynamic driving force for parasitic redox reactions. Analogous to the BTO‐CeO_2_ heterostructure reported by Akbar et al., [56] high‐permittivity dielectrics such as BTO can establish a built‐in electric field at the heterointerface via spontaneous polarization. This interfacial field has been experimentally demonstrated to redistribute charge carriers, suppress electron leakage, and facilitate Li^+^ transport kinetics—effects directly corroborated by our in‐situ DRT analysis and finite‐element simulations.

TEM (Figure 4e) reveals striking differences in interfacial phase structure. The pristine LPSC1.5/NCM83 interface develops a thick amorphous reaction layer (∼10 nm), characteristic of extensive interphase growth. In contrast, LPSC1.5@10BTO retains a much thinner amorphous layer (∼2.5 nm), with the BTO layer still visibly intact. Elemental mapping (Figures S21 and S22) confirms the presence of Ti, Ba, and O at the interface, indicating the physical persistence of the coating. This thinner, stable interphase minimizes Li^+^ transport resistance and explains the enhanced cycling stability [57]. Complementary cross‐sectional SEM images (Figure 4f; Figure S23) show severe cracking and delamination in cycled PCNCM83/LPSC1.5 composites, whereas PCNCM83/LPSC1.5@10BTO retains a dense, well‐adhered morphology with minimal cracking. This structural resilience ensures continuous Li^+^ pathways and mechanical integrity during extended cycling [58, 59].

Together, these multi‐technique analyses elucidate the comprehensive interfacial mechanisms driving the performance of LPSC1.5@10BTO. The BTO nanocoating provides three synergistic advantages: 1) homogenization of interfacial charge transport and Li^+^ flux, 2) suppression of parasitic reactions and byproduct accumulation, and 3) minimization of interfacial decomposition and structural damage. These effects collectively enable rapid charge transfer and ultralong cycling life, establishing the BTO nanocoating as a transformative and scalable interfacial design for high‐performance sulfide‐based ASSBs.

### 2.5 Universality of BTO Surface Engineering and High‐Loading Performance

The versatility of a solid electrolyte across different cathode chemistries is a decisive criterion for its practical deployment [60, 61, 62, 63]. To evaluate the generality of the BTO nanocoating strategy, we investigated the electrochemical performance of BTO‐modified Li‐P‐S‐X electrolytes paired with multiple Ni‐rich cathodes, including PCNCM83, single‐crystal NCM811 (SCNCM811; Figure S24), and SCNCM83 (Figure S25). The tested electrolytes include a commercial Li_7_PS_6_‐based system (LPSC; ionic conductivity 5.17 mS cm^−1^; Figures S26–S28) and a modified Li‐P‐S‐Cl‐Br electrolyte (LPSCB; 12.83 mS cm^−1^; Figures S29–S31). Their BTO‐coated counterparts—LPSC@10BTO and LPSCB@10BTO—retain comparable conductivities of 4.98 and 12.14 mS cm^−1^, respectively, confirming that the coating process preserves ionic transport efficiency.

**FIGURE 5:**
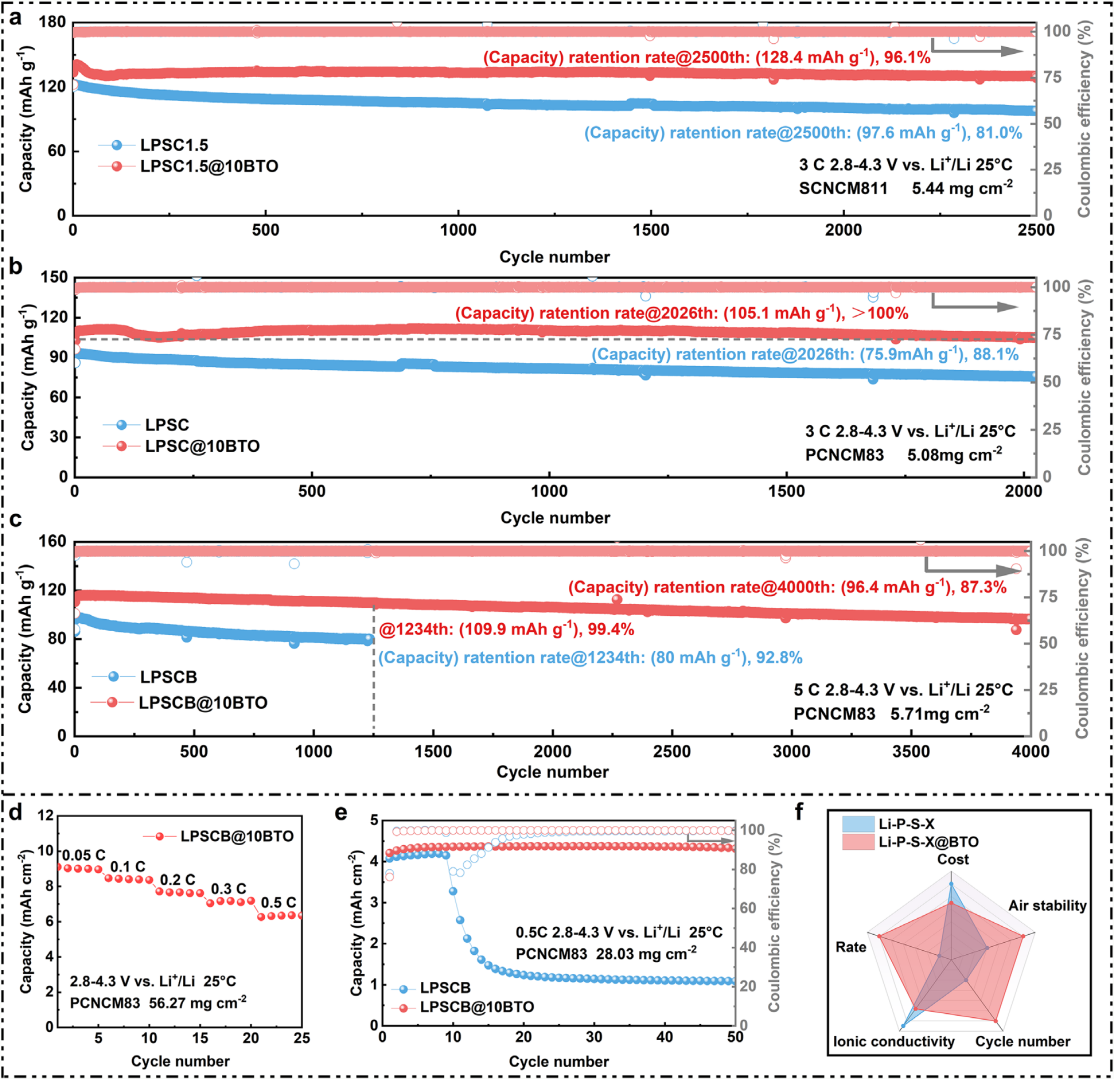
NCM ASSB cells employing BTO coated SSE. (a) Cycle performance of SCNCM811/LPSC1.5@xBTO (x = 0, and 10)/LiIn at 3 C within the voltage range of 2.8–4.3 V vs. Li^+^/Li. (b) Cycle performance of PCNCM83/LPSC@xBTO (x = 0, and 10)/LiIn at 3 C within the voltage range of 2.8–4.3 V vs. Li^+^/Li. (c) Cycle performance of PCNCM83/LPSCB@xBTO (x = 0, and 10)/LiIn at 5 C within the voltage range of 2.8‐4.3 V vs. Li^+^/Li. (d) Rate performance of PCNCM83/LPSCB@10BTO/LiIn at a cathode active material mass loading of 56.27 mg cm^−2^. (e) Cycle performance of PCNCM83/LPSCB@ xBTO (x = 0, and 10)/LiIn at a cathode active material mass loading of 28.03 mg cm^−2^ and 0.5 C. (f) Radar chart illustrating the advantages of the multifunctional nano‐scale BTO coating strategy for Li‐P‐S‐X‐based electrolytes.

For cells employing commercial SCNCM811 cathodes (Figure 5a), LPSC1.5@10BTO delivers a stable discharge capacity of 133.6 mAh g^−1^ at 3 C (2.8‐4.3 V vs. Li^+^/Li, 25°C) and retains 96.1% of its initial capacity (128.4 mAh g^−1^) after 2500 cycles, with Coulombic efficiency consistently exceeding 99.9%. In comparison, pristine LPSC1.5 retains only 81% (97.6 mAh g^−1^) over the same duration. Similar improvements are observed at 1 C (Figure S32) and for SCNCM83 cathodes (Figure S33), underscoring the coating's effectiveness in stabilizing reactive Ni‐rich NCM interfaces. For PCNCM83‐based cells (Figure 5b), LPSC@10BTO achieves 102.6 mAh g^−1^ at 3 C and maintains 102.4% of its initial capacity after 2026 cycles, whereas pristine LPSC shows 88.1% retention (75.9 mAh g^−1^). These results affirm that the BTO nanocoating mitigates interfacial degradation across diverse Ni‐rich cathodes.

Furthermore, under high‐rate operation (5 C, Figure 5c), LPSCB@10BTO/PCNCM83 cells demonstrate exceptional durability, maintaining 87.3% of their capacity (96.4 mAh g^−1^) after 4000 cycles and even 99.4% (109.9 mAh g^−1^) after 1234 cycles. In contrast, pristine LPSCB rapidly declines to 80 mAh g^−1^ and 92.8% retention after 1234 cycles. The robustness of the coating is further confirmed under high cathode active material loadings. At 56.27 mg cm^−2^ (Figure 5d; Figures S34 and S35), LPSCB@10BTO exhibits stable rate capability across 0.05–0.5 C, delivering areal capacities of 9.1, 8.5, 7.7, 7.0, and 6.3 mAh cm^−2^. Even at a moderate cathode active material loading of 28.03 mg cm^−2^ (Figure 5e), LPSCB@10BTO retains 102.5% of its initial capacity after 50 cycles at 0.5 C, compared with only 26.7% for the uncoated electrolyte.

Taken together, these findings confirm the broad applicability of the BTO nanocoating across multiple commercial Li‐P‐S‐X electrolytes (LPSC, LPSC1.5, LPSCB) and Ni‐rich cathodes (PCNCM83, SCNCM811, SCNCM83). Whether tested at high rates (3–10 C), under extended cycling (>10 000 cycles), or at high cathode loadings (>50 mg cm^−2^), BTO‐modified electrolytes consistently outperform their pristine counterparts in both capacity retention and interfacial stability. Combined with their high ionic conductivity, scalability, and strong air stability (Figure 5f), these results establish the multifunctional BTO coating as a practical, universally compatible, and industrially viable engineering strategy for the commercialization of sulfide‐based ASSBs.

## 3 Conclusions

This work presents a scalable BTO nanocoating strategy that addresses the key challenges limiting sulfide‐based ASSBs. Unlike conventional sulfide modifiers such as LiPO_2_F_2_ and LiC_2_O_4_BF_2_, BTO maintains low interfacial impedance even at high loading (15 wt.%), while simultaneously reducing electrolyte cost by ∼8%–12%. The coating markedly enhances air stability, suppressing H_2_S emission by 67%, and enables long‐term cycling stability exceeding 10 000 cycles at 7 C. Beyond these practical advantages, the study provides mechanistic insights into the role of dielectric interfaces in stabilizing solid‐state systems. The BTO layer acts as a multifunctional interfacial modulator—offering physical protection against moisture‐induced degradation and mitigating space‐charge layer formation via its intrinsic polarization. This dual mechanism preserves the structural and electrochemical integrity of both electrolyte and cathode during extended cycling. The demonstrated compatibility across various sulfide electrolytes (LPSC, LPSCB) and cathode chemistries (NCM811, NCM83) underscores the universality of this approach. The simplicity and scalability of the 10 min ball‐milling process position this strategy as a viable pathway toward industrial‐scale production of durable, high‐performance ASSBs.

In future work, we aim to further refine the mechanistic understanding of the BTO nanocoating strategy by integrating the state‐resonant energy transmission law to investigate the Li^+^ energy‐state accessibility mechanism at the interface. This effort is anticipated to establish a novel theoretical framework for interfacial regulation in sulfide‐based ASSBs, paving the way for the rational design of high‐performance electrolyte‐cathode systems.

## Author Contributions

**Wenjin Li** drafted the manuscript, with revisions conducted by **Wenjin Li**, **Qingmei Xiao**, and **Guangliang Gary Liu**. **Guangliang Gary Liu** conceived the project and supervised the research. **Qingmei Xiao**, **Shiming Huang**, **Ruonan Zhang**, and **Hong Yu** were responsible for sample fabrication. **Donghao Liang**, **Kaiyuan Deng**, **Cheng Liu**, **Beisen Chen** and **Puxi An** carried out the experiments and analyzed the corresponding data. All authors participated in the discussion of the results.

## Conflicts of Interest

The authors declare no conflicts of interest.

## Supporting information




**Supporting File**: advs74722‐sup‐0001‐SuppMat.pdf

**Supporting File**: advs74722‐sup‐0002‐VideoS1.mp4

**Supporting File**: advs74722‐sup‐0003‐VideoS2.mp4

## Data Availability

The data that support the findings of this study are available on request from the corresponding author. The data are not publicly available due to privacy or ethical restrictions.
