# Supplementary material for: A Scalable BaTiO3 Nanocoating Strategy for Cost‐Effective and Stable Sulfide‐Based All‐Solid‐State Batteries
Source: Adv Sci (Weinh). 2026 Mar 4;13(28):e74722. doi: 10.1002/advs.74722 (PMC13185824; doi:10.1002/advs.74722)
Supplement: Supplementary file 1 — Supporting File: advs74722‐sup‐0001‐SuppMat.pdf [file ADVS-13-e74722-s001.docx]

Supporting Information

**A scalable BaTiO_3_ nanocoating strategy for cost-effective and stable sulfide-based all-solid-state batteries**

*Wenjin Li^#^, Qingmei Xiao^#^,* *Shiming Huang, Ruonan Zhang, Hong Yu, Donghao Liang, Kaiyuan Deng, Cheng Liu, Beisen Chen, Puxi An, Guangliang Gary Liu**

Guangdong Provincial Key Laboratory of New Energy Materials Service Safety

College of Materials Science and Engineering

Shenzhen University

Shenzhen, 518060, China

*Corresponding author E-mail: ggliu@szu.edu.cn (Prof. G. Liu)

^#^Wenjin Li and Qingmei Xiao contributed equally to this work

1. Experimental Section

***1.1. Materials Preparation***

**Synthesis of Pristine Sulfide Electrolytes**

Pristine Li_5.5_PS_4.5_Cl_1.5_ (LPSC1.5) was synthesized from high-purity Li_2_S, P_2_S_5_, and LiCl precursors. Stoichiometric amounts of the starting materials were manually pre-mixed in an agate mortar for 5 min and subsequently transferred into a zirconia jar with 5-mm zirconia milling media at a media-to-powder mass ratio of 40:1. The mixture was milled in a planetary ball mill (Changsha Miqi Instrument & Equipment Co., Ltd.) at 550 rpm for 30 min under an argon atmosphere using a cyclic program of 10 min counterclockwise rotation followed by 6 min rest. The resulting precursor powder was pressed into pellets, sealed in an alumina crucible, and annealed at 450 °C for 12 h under flowing argon to yield pristine LPSC1.5. Commercial Li_5.5_PS_4.5_Cl_0.8_Br_0.7_ (LPSCB) and Li_6_PS_5_Cl (LPSC) electrolytes were purchased from Shenzhen Yanpin Technology Co., Ltd. and Shenzhen Kejing Technology Co., Ltd., respectively. Commercial BaTiO_3_ (BTO) nanoparticles (99.9% purity, Buwei Co., Ltd.) were employed as the dielectric coating material.

**Preparation of BaTiO_3_-Modified Electrolytes**

Modified sulfide electrolytes were fabricated by high-energy ball milling (Changsha Miqi Instrument & Equipment Co., Ltd.). For LPSC1.5-based composites, BTO nanoparticles were mixed with LPSC1.5 at mass fractions of 0, 5, 10, and 15 wt%. The mixtures were milled at 325 rpm for 10 min using zirconia grinding media at a media-to-powder mass ratio of 30:1. The resulting products were denoted as LPSC1.5, LPSC1.5@5BTO, LPSC1.5@10BTO, and LPSC1.5@15BTO, respectively. For LPSC and LPSCB systems, a fixed BTO content of 10 wt% was adopted under identical milling conditions (325 rpm, 10 min, media-to-powder ratio of 30:1). The corresponding products were labeled as LPSC@10BTO and LPSCB@10BTO.

**Synthesis of Polycrystalline NCM83 Cathode**

Polycrystalline LiNi_0.83_Co_0.11_Mn_0.06_O_2_ (PCNCM83) was synthesized via solid-state reaction. A hydroxide precursor, Ni_0.83_Co_0.11_Mn_0.06_(OH)_2_ (3-5 µm particle size, Hunan Shanshan Co., Ltd.), was mixed with LiOH·H_2_O (Ganfeng Lithium Co., Ltd.) at a molar ratio of 1.08:1 (Li: transition metals). The homogenized mixture was first calcined at 480 °C for 5 h, followed by a second annealing step at 780 °C for 12 h under continuous oxygen flow. The final powder was collected and designated as PCNCM83.

***1.2. Moisture Stability Test***

Humidity tolerance measurements were performed using a custom-built detection chamber supplied with flowing N₂ gas at controlled relative humidity levels of 30 ± 5% and 70 ± 5% at 25 °C. Sulfide solid electrolyte (SSE) powders or pellets were exposed to these conditions, and the evolution of H₂S gas was continuously monitored using a gas detector (JES-MS400-H₂S-RD). For quantitative comparison, 200 mg of each SSE sample was pressed into pellets with identical surface areas to ensure consistent exposure and reliable evaluation of moisture-induced degradation behavior.

***1.3. Materials Characterization***

The morphological evolution of SSE particles was examined using an optical microscope (Olympus DSX1000), and particle size distributions were quantified using Nano Measurer software. Morphological and microstructural analyses were carried out with a field-emission scanning electron microscope (FE-SEM, Hitachi SU-70) equipped with an energy-dispersive X-ray spectroscopy (EDS) detector. For cross-sectional SEM observations, discharged electrodes were disassembled in an argon-filled glovebox and subsequently polished using an argon ion beam milling system (JEOL IB-19520CCP). Atomic-scale microstructural features were further examined by transmission electron microscopy (TEM, JEOL F200) operated at 200 kV.

Phase identification and crystal structure analyses were performed using an X-ray diffractometer (Rigaku SmartLab) with Cu Kα radiation (λ = 1.5406 Å, 40 kV, 30 mA) over a 2θ range of 10°-90° at a scanning rate of 5° min^-1^. Elemental composition and chemical states were analyzed by X-ray photoelectron spectroscopy (XPS, Thermo ESCALAB 250) employing a monochromatic Al Kα source (1486.6 eV). Time-of-flight secondary ion mass spectrometry (ToF-SIMS) was conducted on a TOF-SIMS 5-100 instrument (IONTOF). All sample preparation was carried out in an argon-filled glovebox. Composite cathodes were mounted on sample holders using insulating adhesive tape and transferred to the analysis chamber under inert conditions via a Leica EM VCT500 vacuum transfer system (Leica Microsystems). ToF-SIMS measurements were performed in negative ion mode using 25 keV Bi^3+^ primary ions with low-energy electron flooding for charge compensation. The analysis employed a 60 µs cycle time and was conducted in spectrometry (bunched) mode to ensure high signal intensity and mass resolution.

Electrochemical atomic force microscopy (EC-AFM) was conducted in PeakForce TUNA mode (Bruker Dimension Icon) to simultaneously obtain nanoscale current and mechanical property maps. A Pt/Ir-coated Si cantilever (SCM-PIT-V2, Bruker) with a nominal radius of 25 nm, spring constant of 3.0 N m^-1^, and resonance frequency of 75 kHz was used. Current mapping was performed over 5 µm × 5 µm areas at a scan rate of 0.8 Hz (≈3 min per image) with a PeakForce setpoint of ~10 nN, feedback gain of ~15, and oscillation amplitude of 150 nm (1 kHz). The calibrated current sensitivity was approximately 20 pA V^-1^. All EC-AFM measurements were performed inside an argon glovebox to prevent sample degradation by air exposure.

***1.4. Ion Conductivity and Electrochemical Measurements***

**Ionic Conductivity Measurement**

The ionic conductivity of the SSEs was measured via electrochemical impedance spectroscopy (EIS). SSE powders were pressed into pellets (10 mm in diameter) and sandwiched between two stainless steel blocking electrodes inside an argon-filled glovebox. EIS measurements were conducted using a Princeton Applied Research electrochemical workstation (PARSTAT3000A-DX) over a frequency range of 1 MHz to 10 mHz with an applied voltage amplitude of 10 mV. Tests were performed at temperatures from 0 to 100 °C in 10 °C increments. The ionic conductivity (σ) and activation energy (Eₐ) were calculated according to the following equations:

$\sigma=\frac{d}{R_{total}S}$

where *d* is the pellet thickness, *S* is the effective electrode area (0.0785 cm^2^), and *R_total_* is the total resistance obtained from Nyquist plots.

$$E_{a}=KTln( \frac{\sigma}{A})$$

here *A* is the pre-exponential factor, *T* is the absolute temperature (K), and *K* is the Boltzmann constant (1.380649 × 10^-23^ J K^-1^).

**All-Solid-State Battery (ASSB) Fabrication**

Cathode composites were prepared by manually grinding 70 mg of cathode active material with 30 mg of SSE powder in an agate mortar. The cathode materials used included polycrystalline LiNi_0.83_Co_0.11_Mn_0.06_O_2_ (PCNCM83, lab-synthesized), and single-crystal LiNi_0.8_Mn_0.1_Co_0.1_O_2_ (SCNCM811) and LiNi_0.83_Co_0.11_Mn_0.06_O_2_ (SCNCM83), both purchased from Shenzhen Kejing Technology Co., Ltd. For cell assembly, 100 mg of SSE was loaded into a 10 mm stainless steel die and cold-pressed at 150 MPa to form a dense electrolyte pellet. Approximately 6-8 mg of cathode composite was uniformly spread on one side of the pellet and cold-pressed at 300 MPa for 1 min to ensure intimate contact. A Li-In alloy anode was subsequently pressed onto the opposite side at 150 MPa for 3 min. Aluminum and copper foils served as the positive and negative current collectors, respectively. All assembly and sealing steps were carried out under an inert argon atmosphere.

**Electrochemical Testing**

The cyclic voltammetry (CV) curve was measured using electrochemical working station (Solartron 1260) with asymmetric LiIn|SE|SE+VGCF cell at a scan rate of 0.1 mV s^-1^ between 2.5 and 4.3 V vs. Li⁺/Li at 25℃ to obtain the electrochemical window of the LPSC1.5 and LPSC1.5@10BTO electrolyte. Standard galvanostatic charge-discharge measurements were performed using a LAND battery test system within the voltage range of 2.8-4.3 V vs. Li⁺/Li. The cathode active material loading was typically maintained at 5-7 mg cm^-2^, and cycling rates varied from 0.1 C to 10 C (1 C = 200 mA g^-1^). For high-mass-loading tests (20-57 mg cm^-2^), the charge-discharge rates were adjusted to 0.05 C, 0.1 C, 0.2 C, 0.3 C and 0.5 C, where 1 C also corresponds to a current density of 200 mA g^-1^. Lithium-ion diffusion coefficients were determined through galvanostatic intermittent titration technique (GITT) tests, conducted at 0.1 C between 2.8 and 4.3 V vs. Li^+^/Li, with a titration interval of 40 min and a relaxation period of 1 h. In situ EIS measurements were conducted on the ASSBs using a PARSTAT 3000A-DX potentiostat/galvanostat over a frequency range of 1 MHz to 100 mHz throughout the first electrochemical cycle. Cells were cycled at a constant current rate of 0.1 C for 1 h per step, followed by a 30-minute open-circuit rest period to ensure thermodynamic and interfacial equilibration prior to each EIS acquisition. Distribution of relaxation times (DRT) analysis was performed using a validated MATLAB-based graphical user interface (GUI) toolbox to quantitatively resolve the underlying relaxation processes embedded in the EIS spectra.

***1.5. Finite Element Simulations***

A two-dimensional finite element model was constructed in COMSOL Multiphysics 6.3 to simulate the electric field homogenization induced by BTO particles under an applied electric field. The model was based on Poisson’s equation (*E* = −∇*V*), where *E* denotes the electric field and *V* represents the electric potential within the computational domain. The piezoelectric properties of BTO were incorporated into the simulation framework to account for dielectric polarization effects. The computational domain was designed as a rectangular region containing uniformly distributed cubic BTO particles embedded in a dielectric matrix. Boundary conditions were defined such that a potential of 4.3 V was applied to the bottom edge, while all other boundaries were set as electrically insulated. A stationary study using a fully coupled direct solver was employed to obtain the steady-state electric potential and field distributions across the domain.

2. Supplementary Figures


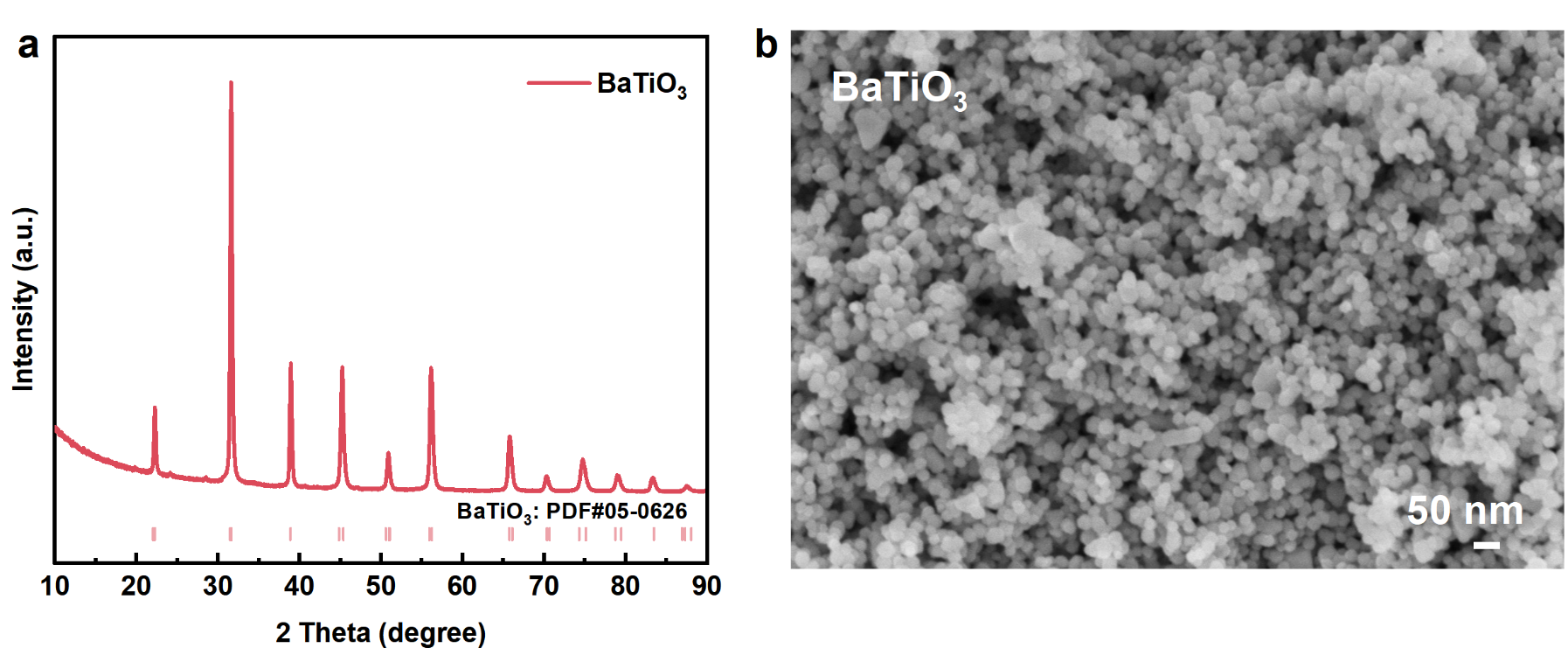


**Figure S1.** (a) XRD patterns and (b) SEM image of BaTiO_3_.


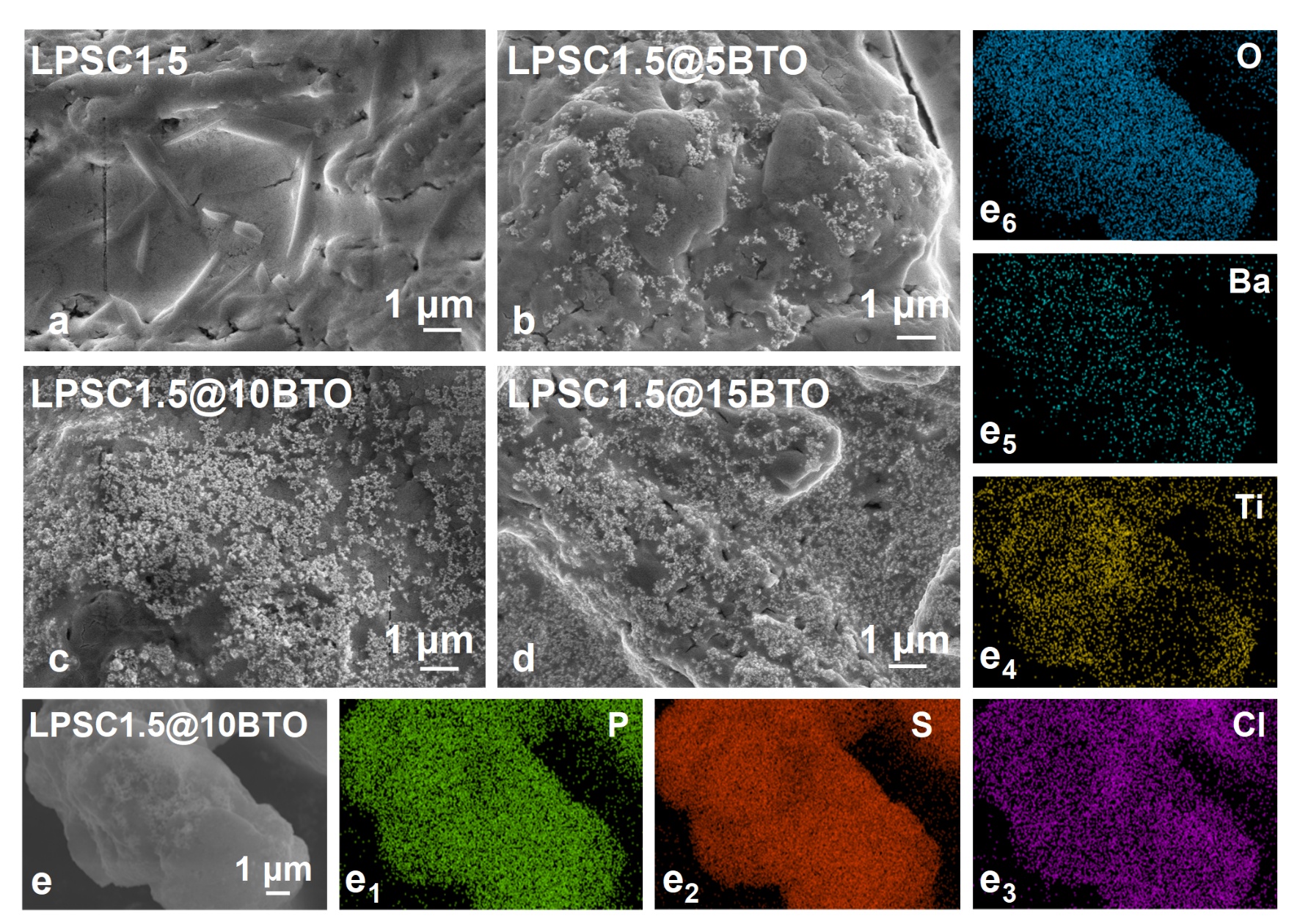


**Figure S2.** SEM images of LPSC1.5@xBTO (a) x=0, (b) x=5, (c) x=10, and (d) x=15. (e) SEM image of LPSC1.5@10BTO with corresponding EDS elemental mapping of (e1-e6) P, S, Cl, Ti, Ba, and O, respectively.

**
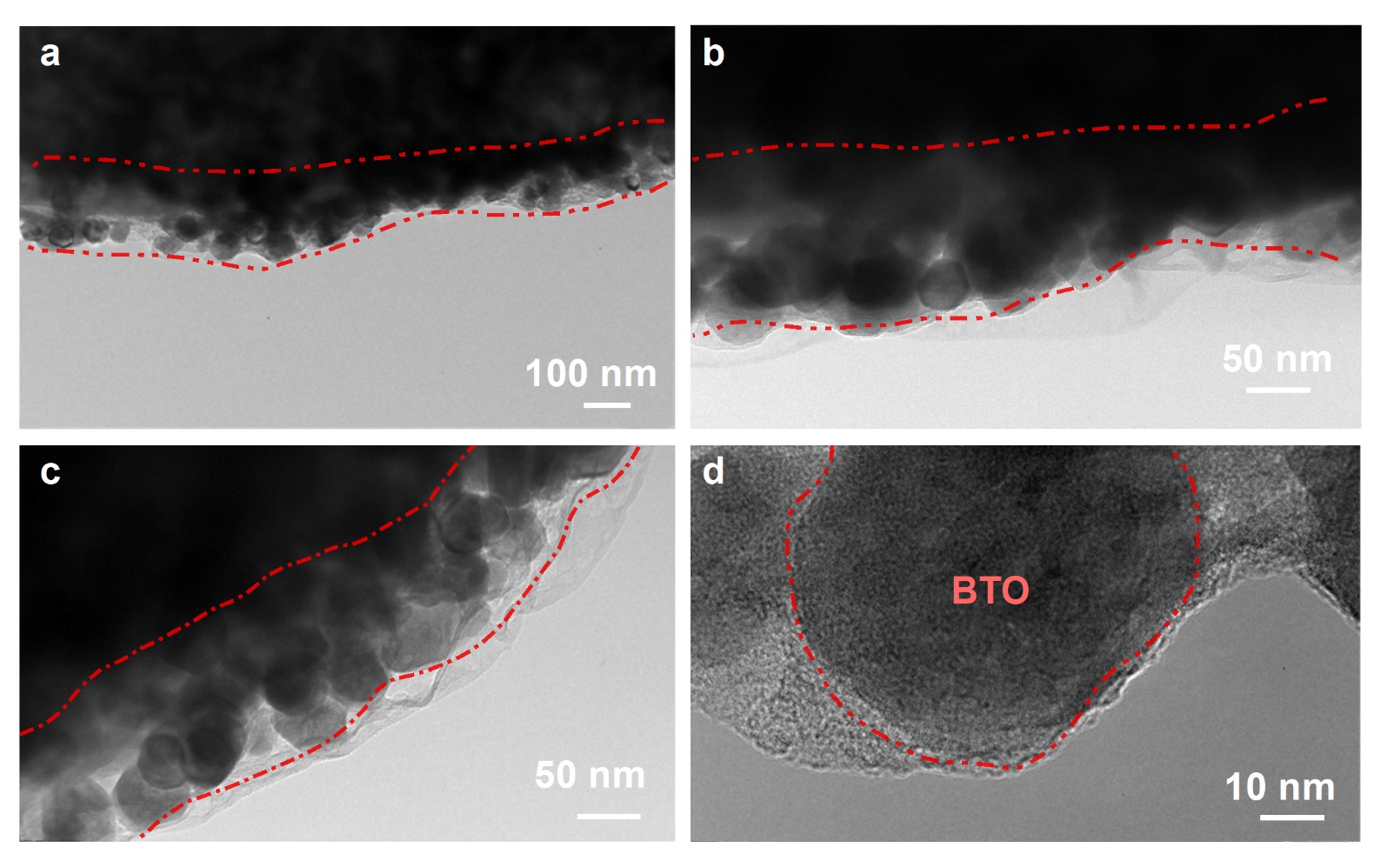
**

**Figure S3.** (a-d) TEM images of LPSC1.5@10BTO at different magnifications.


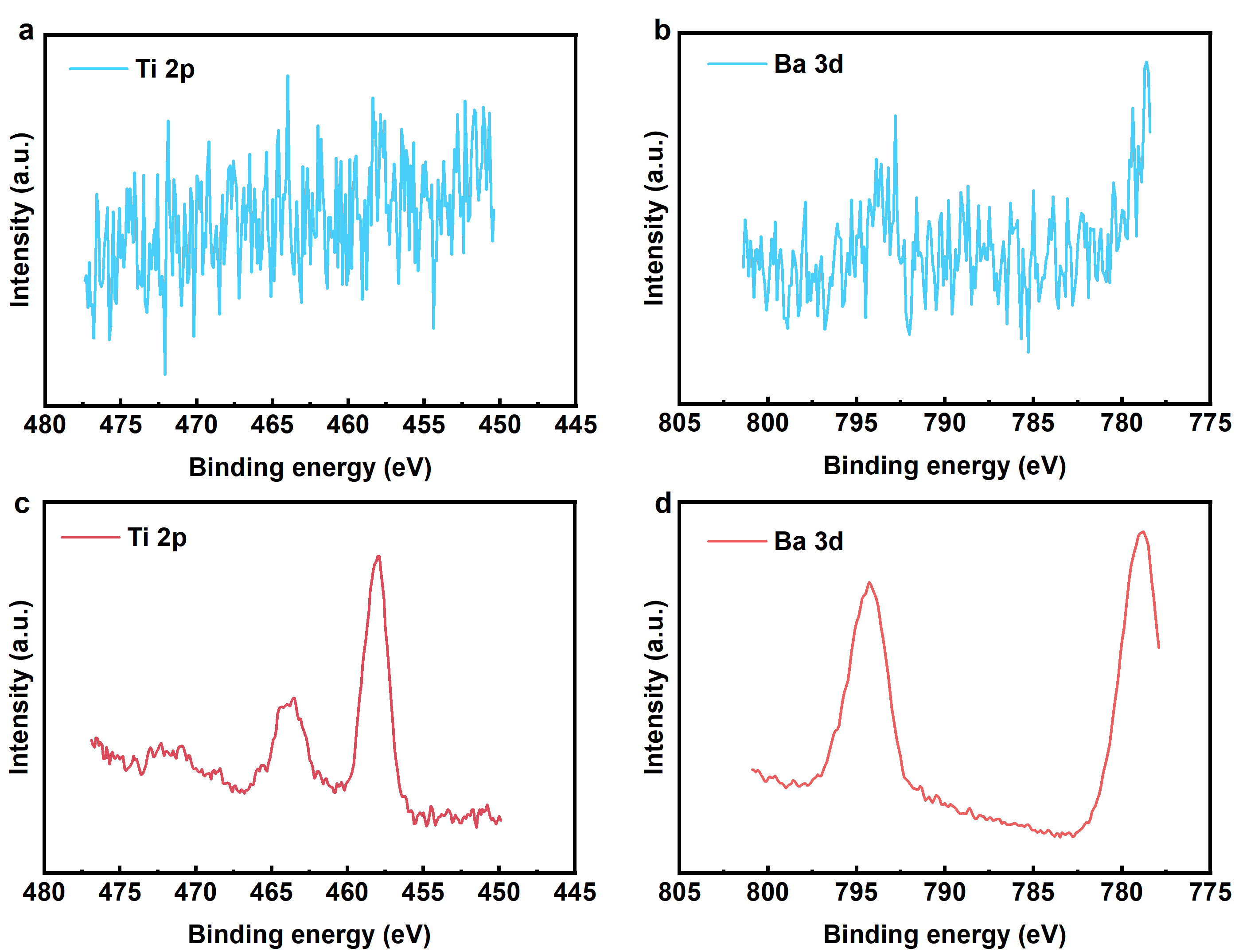


**Figure S4.** XPS characterization of Ti 2p, Ba 3d for (a, b) LPSC1.5 and (c, d) LPSC1.5@10BTO.


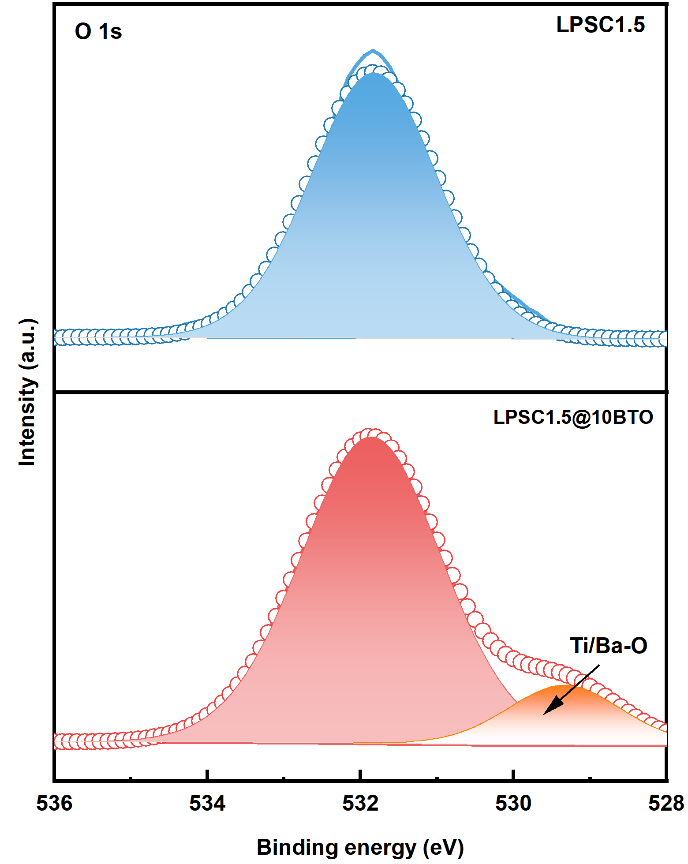


**Figure S5.** XPS characterization of O 1s for LPSC1.5 and LPSC1.5@10BTO.


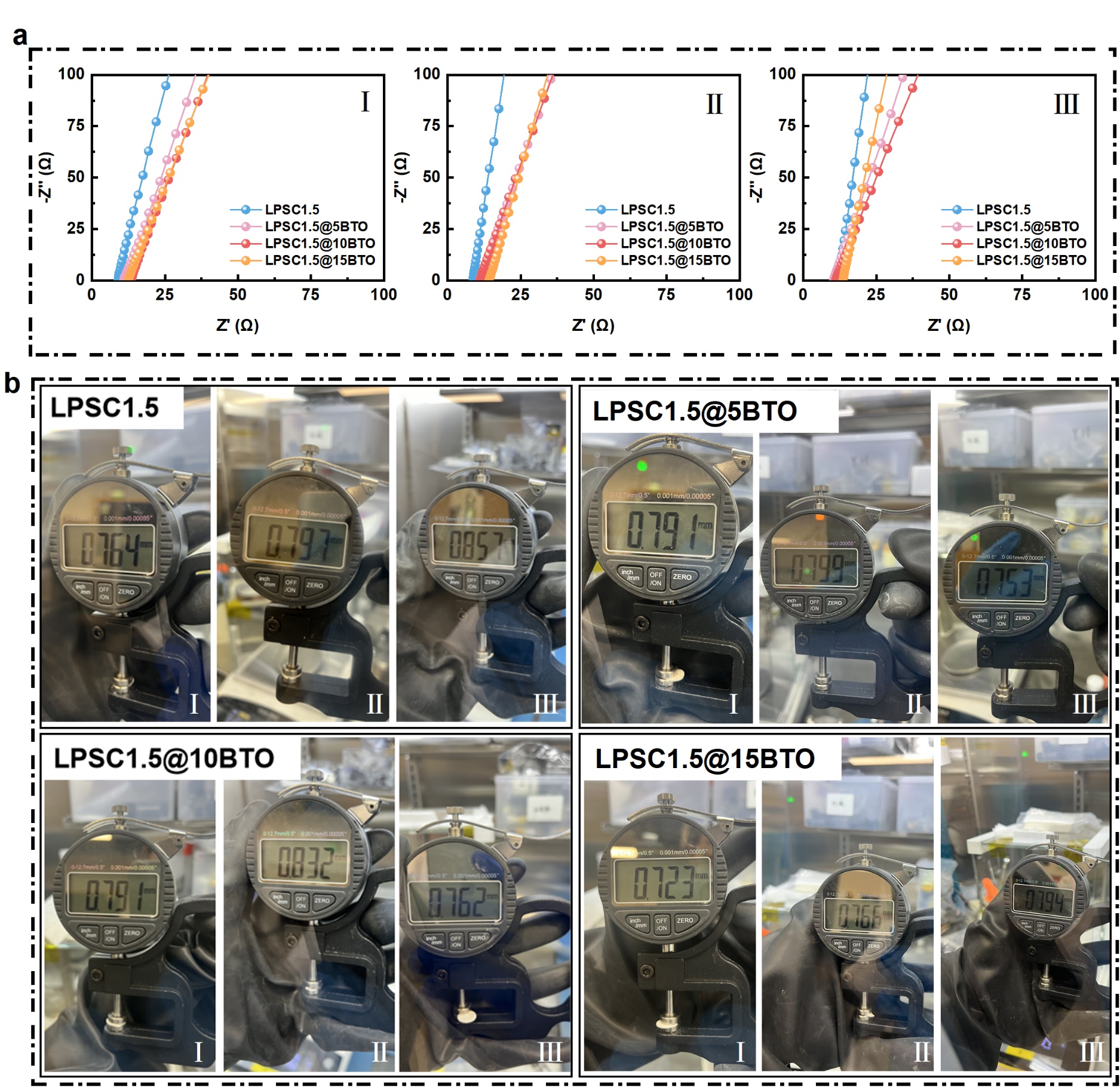


**Figure S6.** (a) The Nyquist plots of LPSC1.5@xBTO (x = 0, 5, 10, 15) measured at 25 ℃. (b) Schematic diagram of measured thickness for LPSC1.5@xBTO (x = 0, 5, 10, 15) pellets.


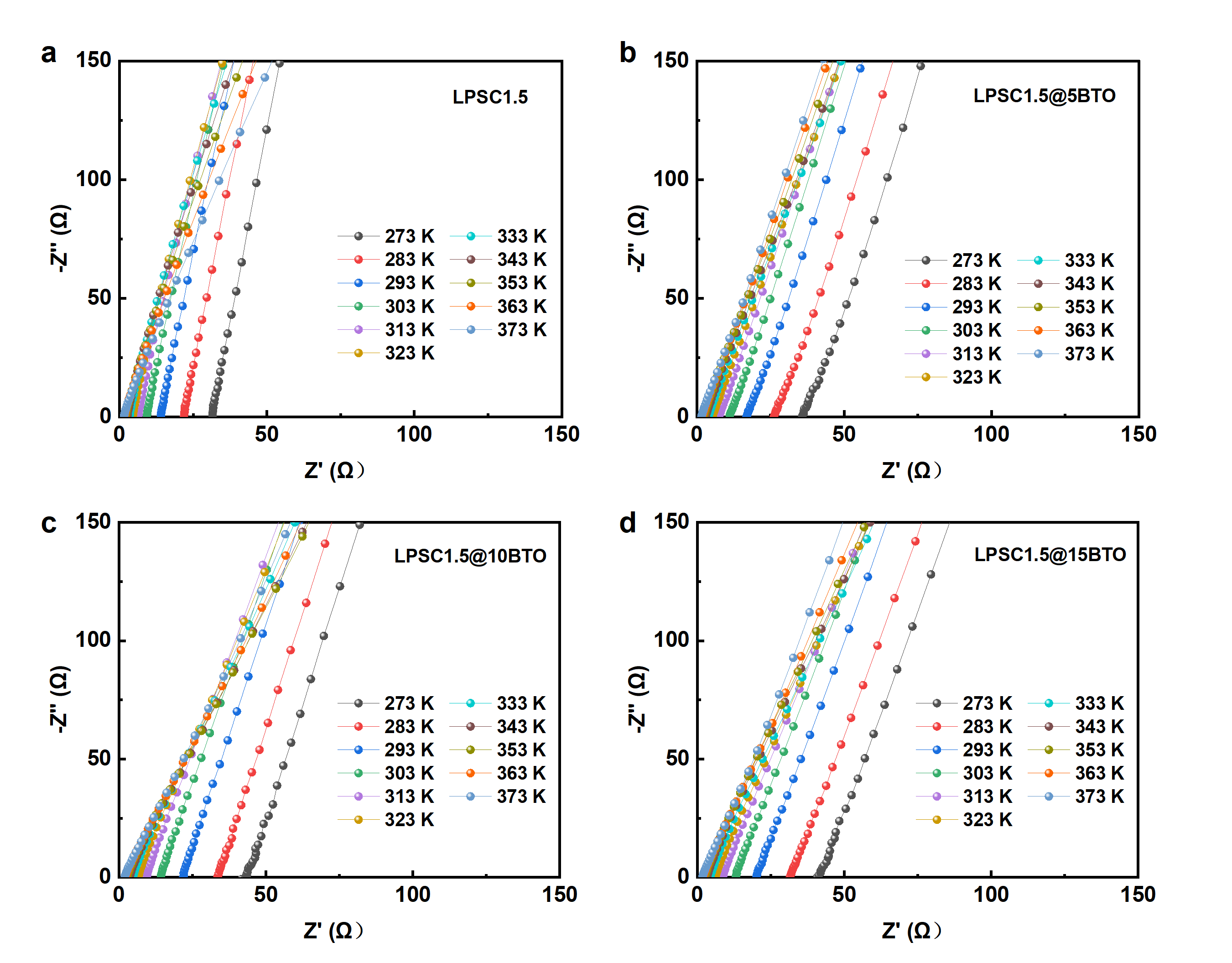


**Figure S7.** The Nyquist plots of (a) LPSC1.5, (b) LPSC1.5@5BTO, (c) LPSC1.5@10BTO, and (d) LPSC1.5@15BTO measured over the temperature range of 0 ℃ to 100 ℃.


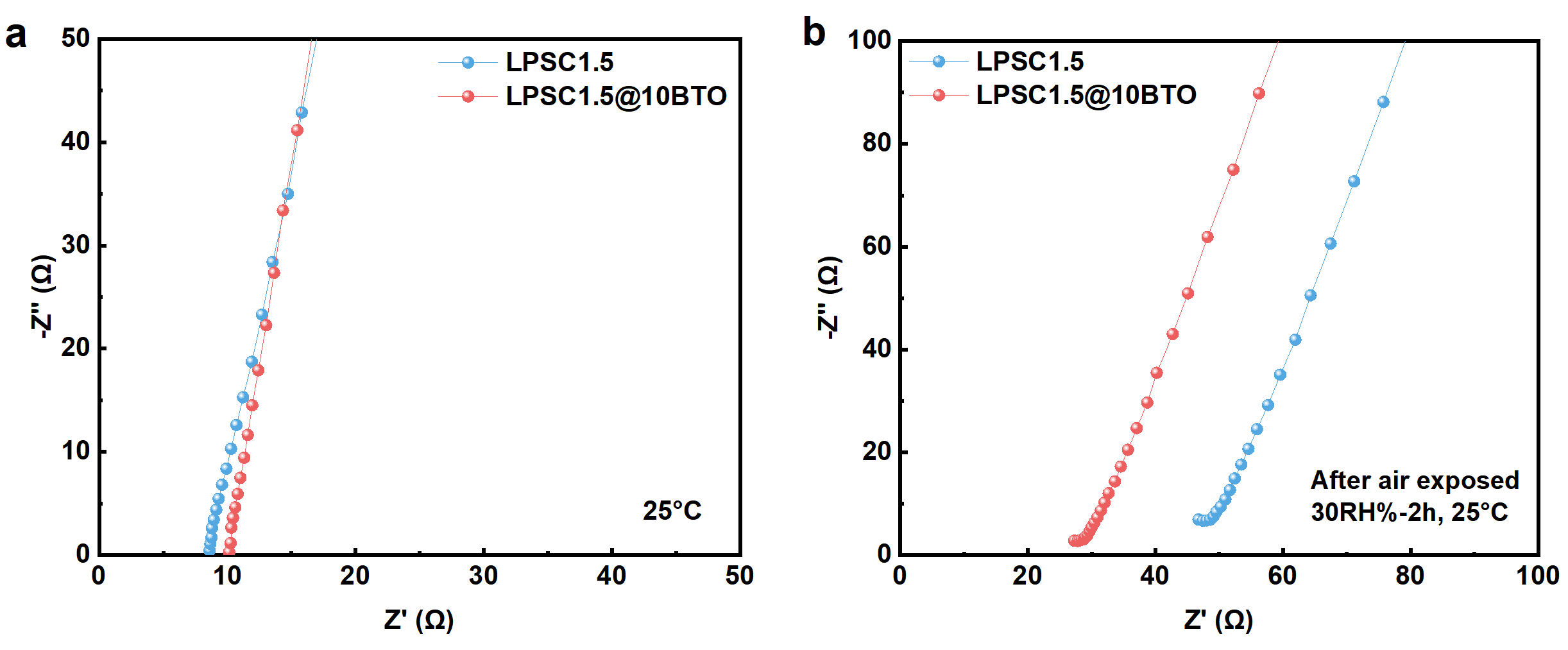


**Figure S8.** The Nyquist plots of LPSC1.5 and LPSC1.5@10BTO pellets (a) before and (b) after exposure to humid air (25 °C, 30 ± 5% RH) for 2 h.


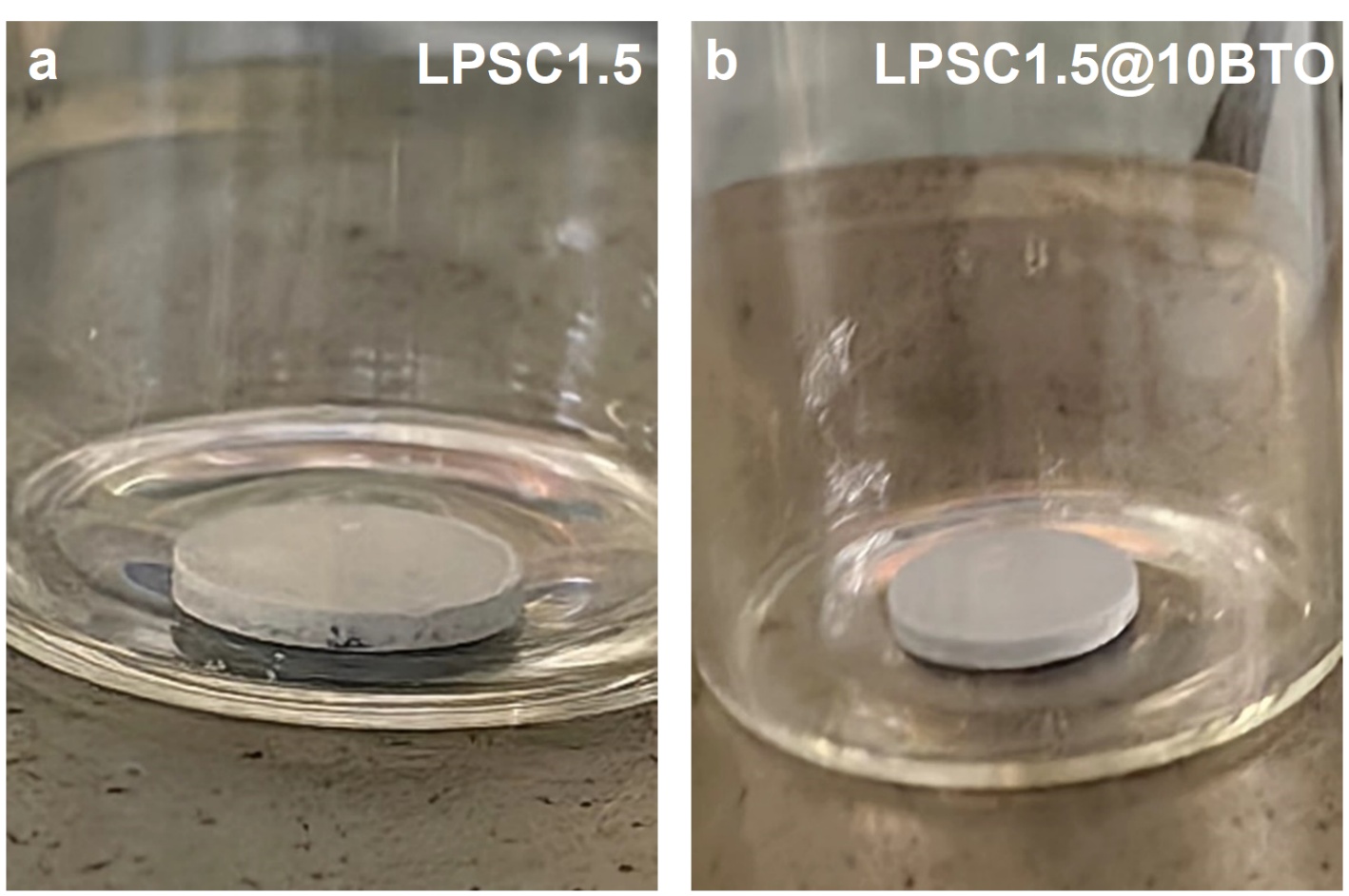


**Figure S9.** Digital photograph of LPSC1.5 and LPSC1.5@10BTO pellets following exposure to humid air (25 °C, 30 ± 5% RH) for 2 h.


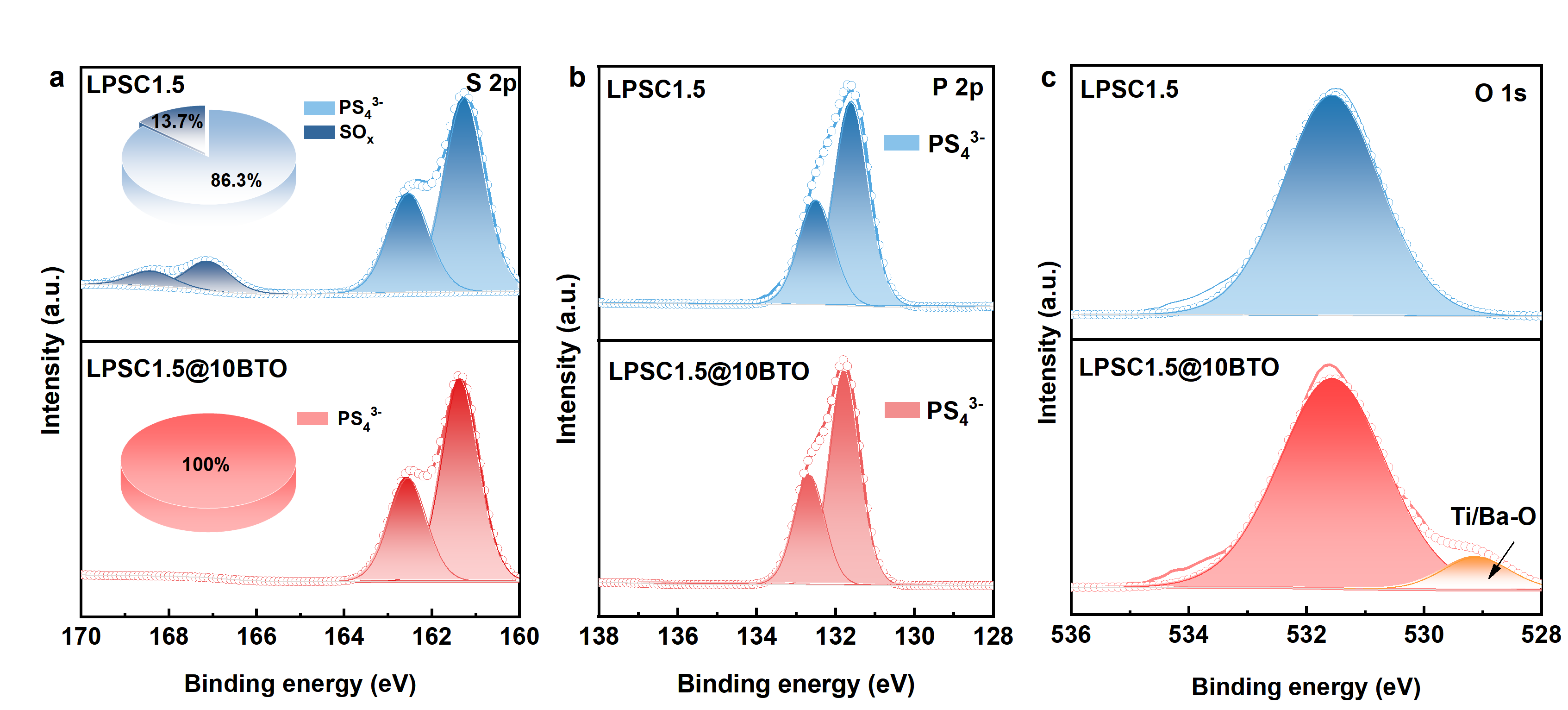


**Figure S10.** XPS characterization of (a) S 2p, (b) P 2p, and (c) O 1s of LPSC1.5 and LPSC1.5@10BTO after exposure to humid air (25 °C, 30 ± 5% RH) for 2 h.


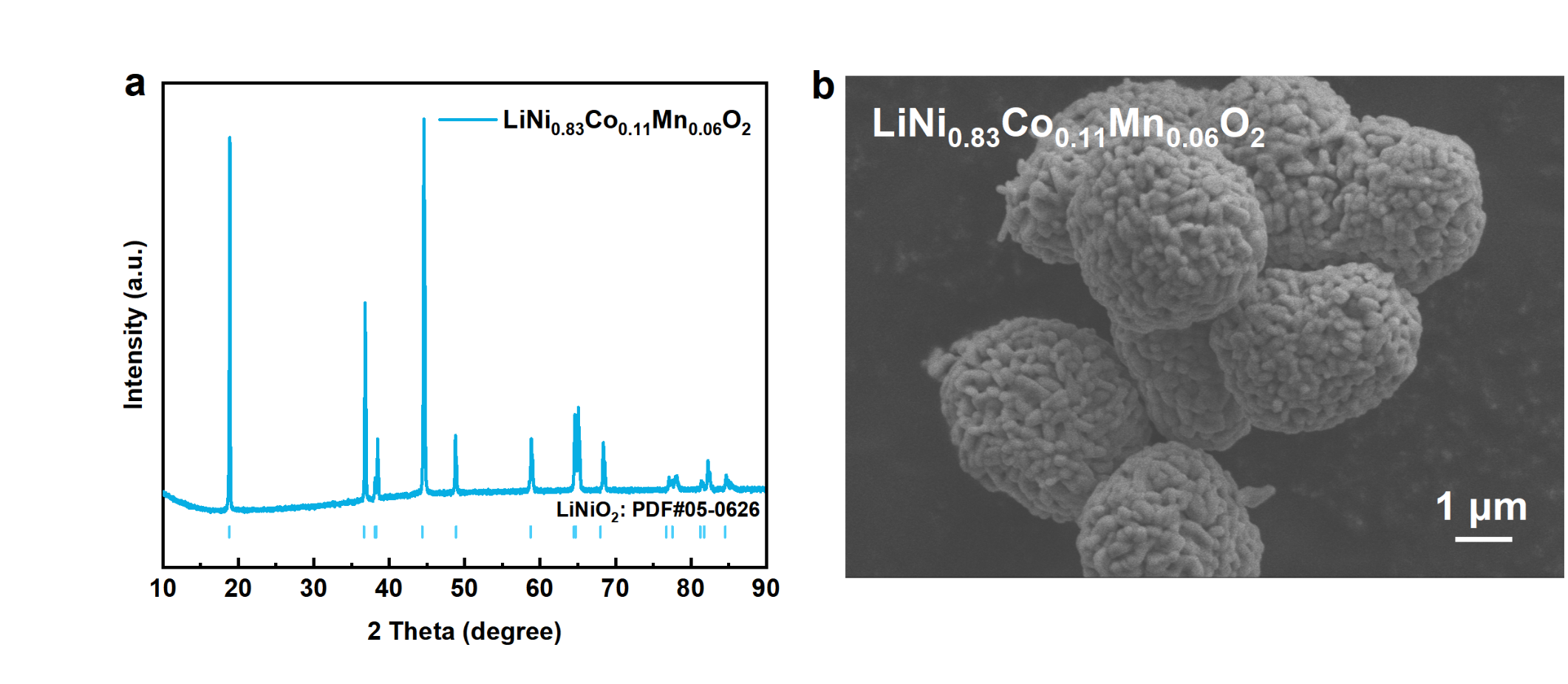


**Figure S11.** (a) XRD pattern and (b) SEM image of PCNCM83.


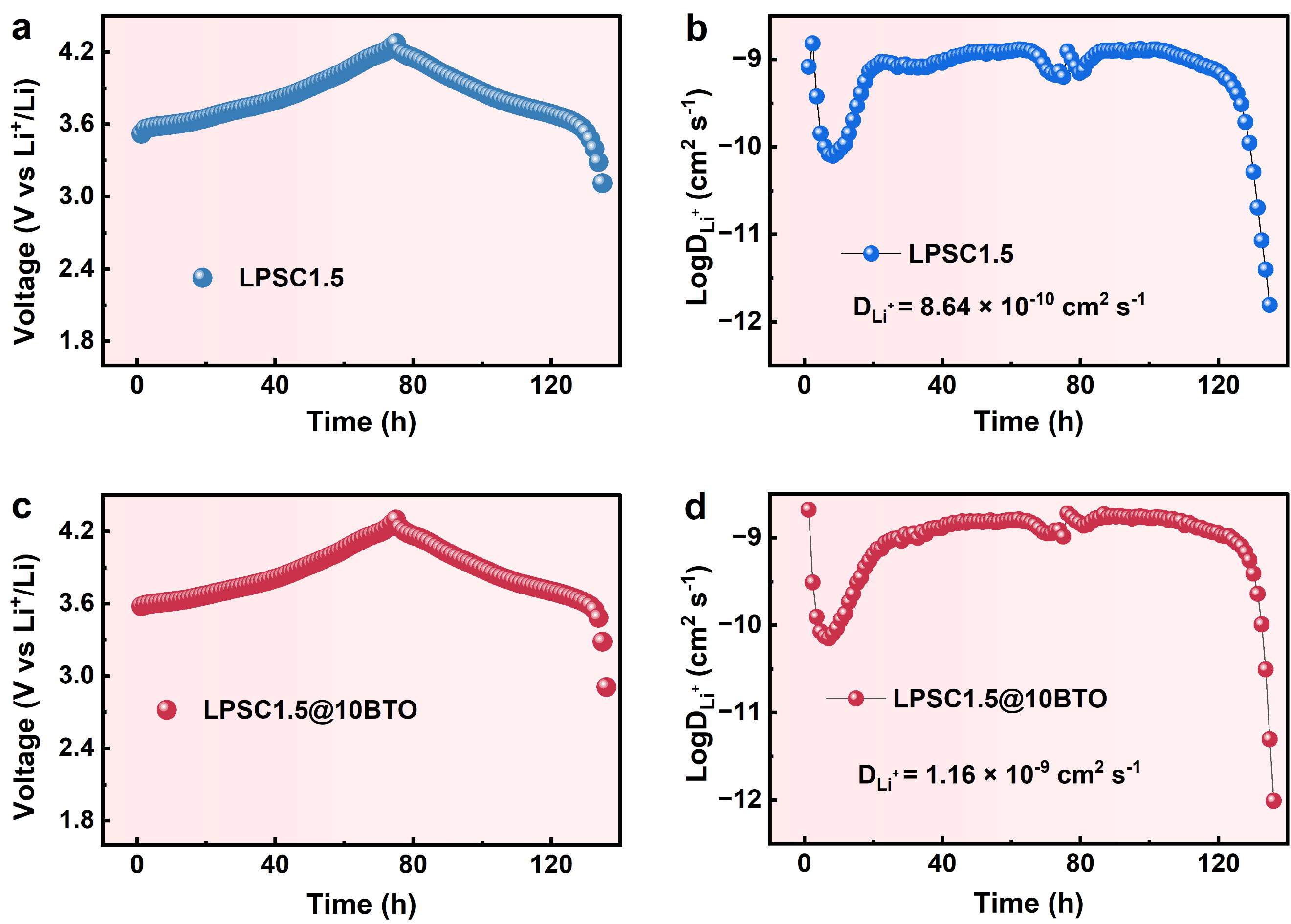


**Figure S12.** Galvanostatic intermittent titration technique (GITT) voltage-time response curves of (a) pristine LPSC1.5 and (c) LPSC1.5@10BTO-based ASSBs. The calculated Li^+^ diffusion coefficient (D_Li_^+^) of (b) pristine LPSC1.5 and (d) LPSC1.5@10BTO-based ASSBs.


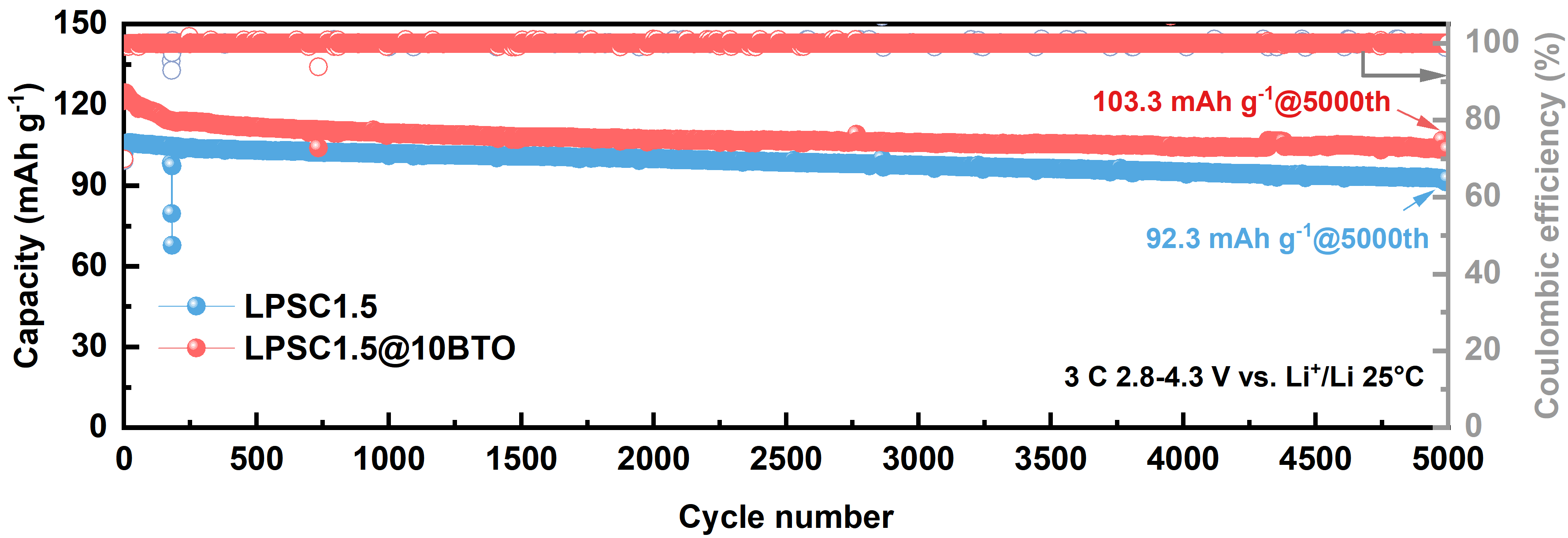


**Figure S13.** Long-term cycling performance of LPSC1.5 and LPSC1.5@10BTO-based ASSBs at 3 C within the voltage range of 2.8-4.3 V vs. Li^+^/Li at 25 ℃.


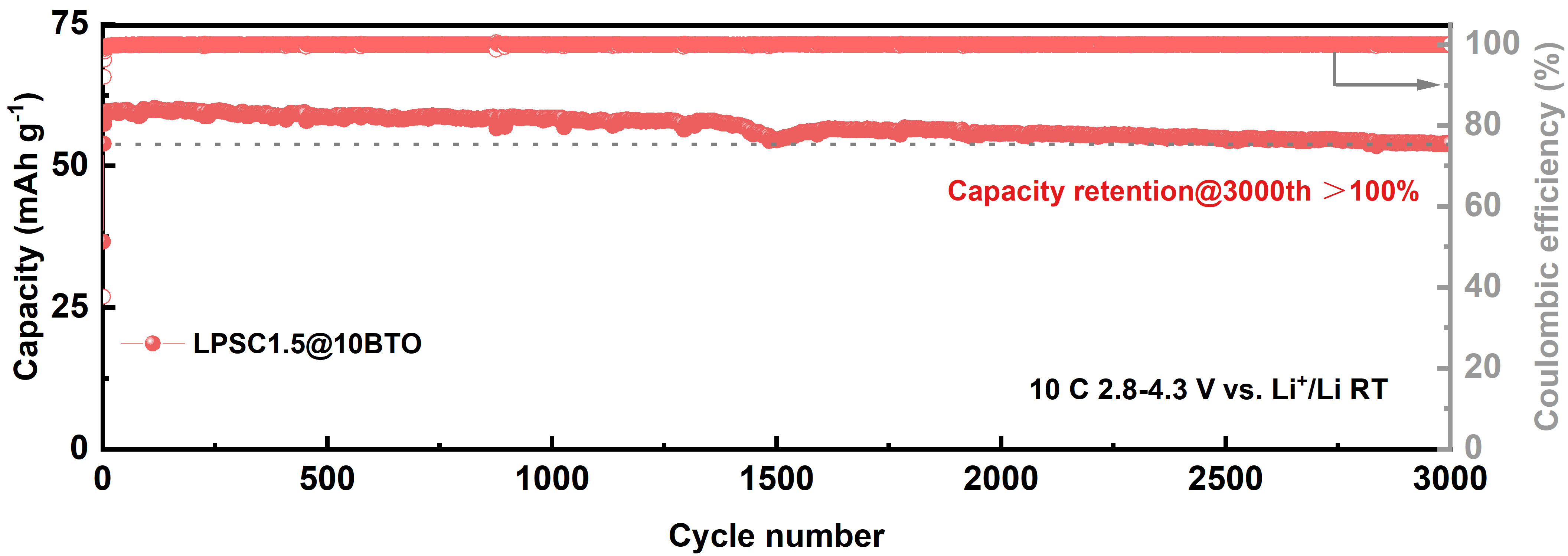


**Figure S14.** Long-term cycling performance of LPSC1.5@10BTO-based ASSBs at 10 C within the voltage range of 2.8-4.3 V vs. Li^+^/Li at 25 ℃.


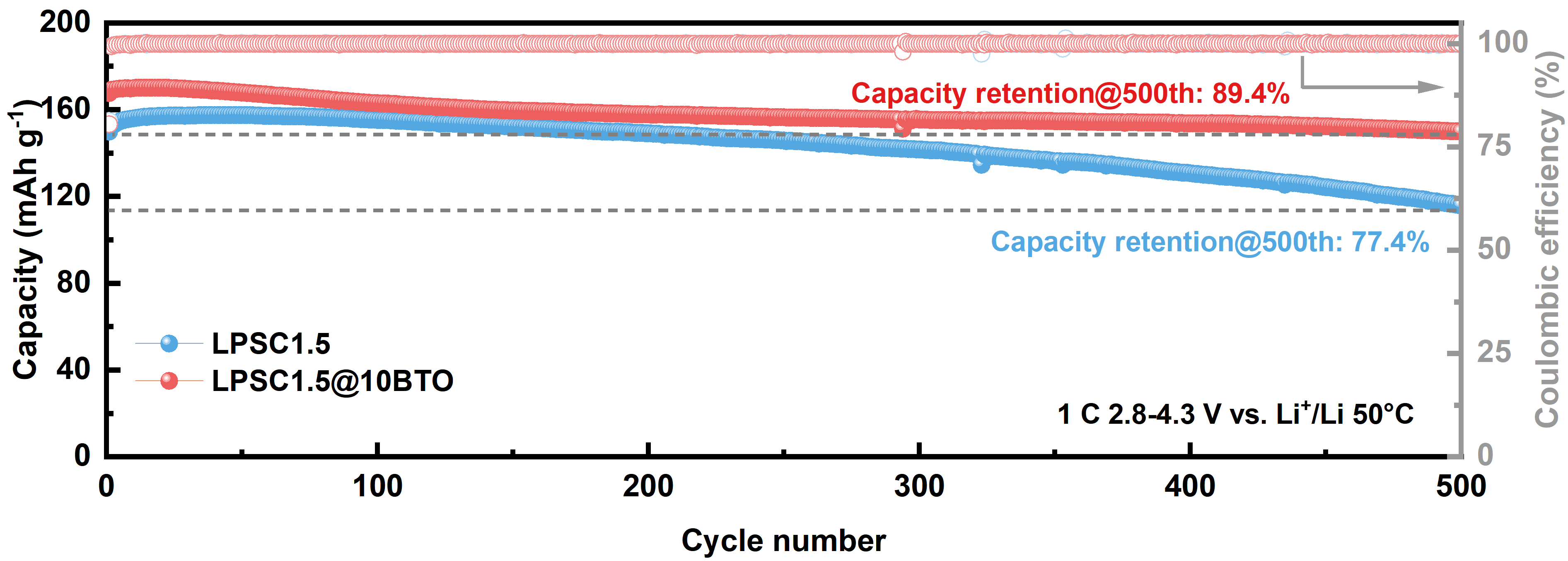


**Figure S15.** Long-term cycling performance of LPSC1.5 and LPSC1.5@10BTO-based ASSBs at 1 C within the voltage range of 2.8-4.3 V vs. Li^+^/Li at 50 ℃.


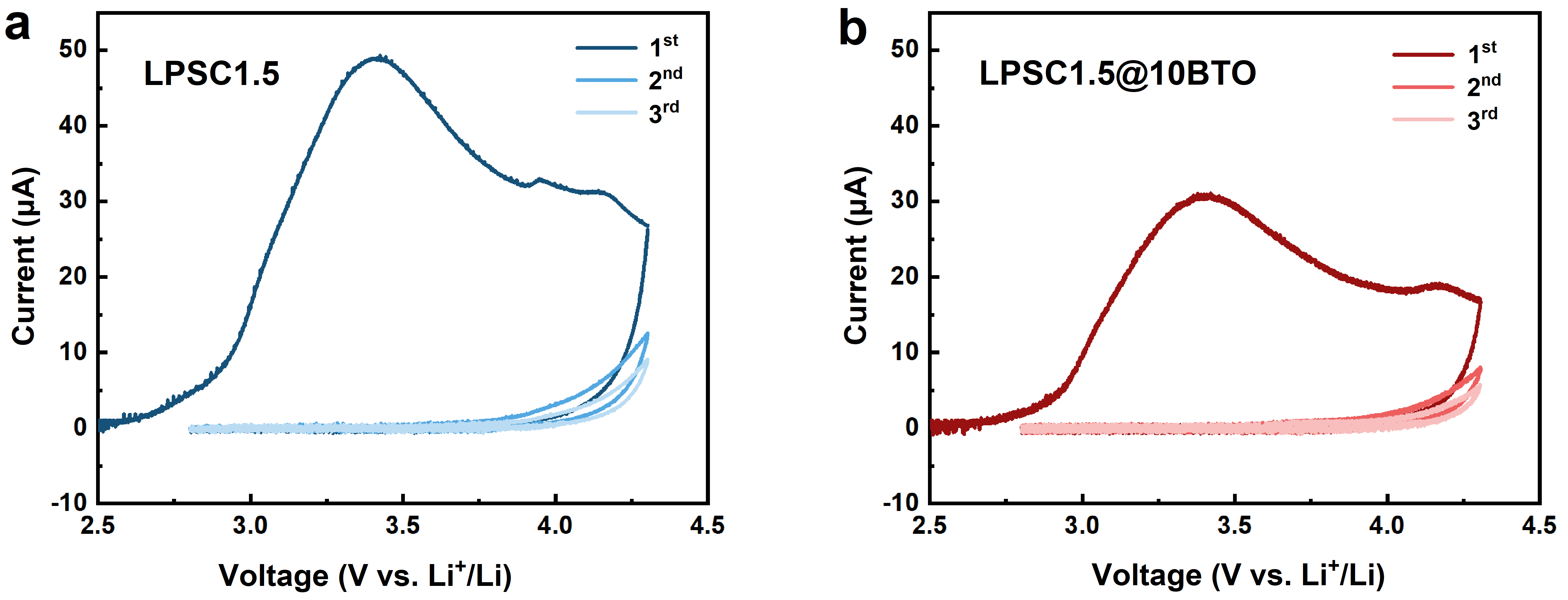


**Figure S16.** Cyclic voltammetry (CV) curves of LiIn|SE|SE+VGCF cells with (a) pristine LPSC1.5 and (b) LPSC1.5@10BTO electrolytes. The tests were conducted at 25 °C with a scan rate of 0.1 mV s^-1^ within the voltage range of 2.5-4.3 V vs. Li^+^/Li.


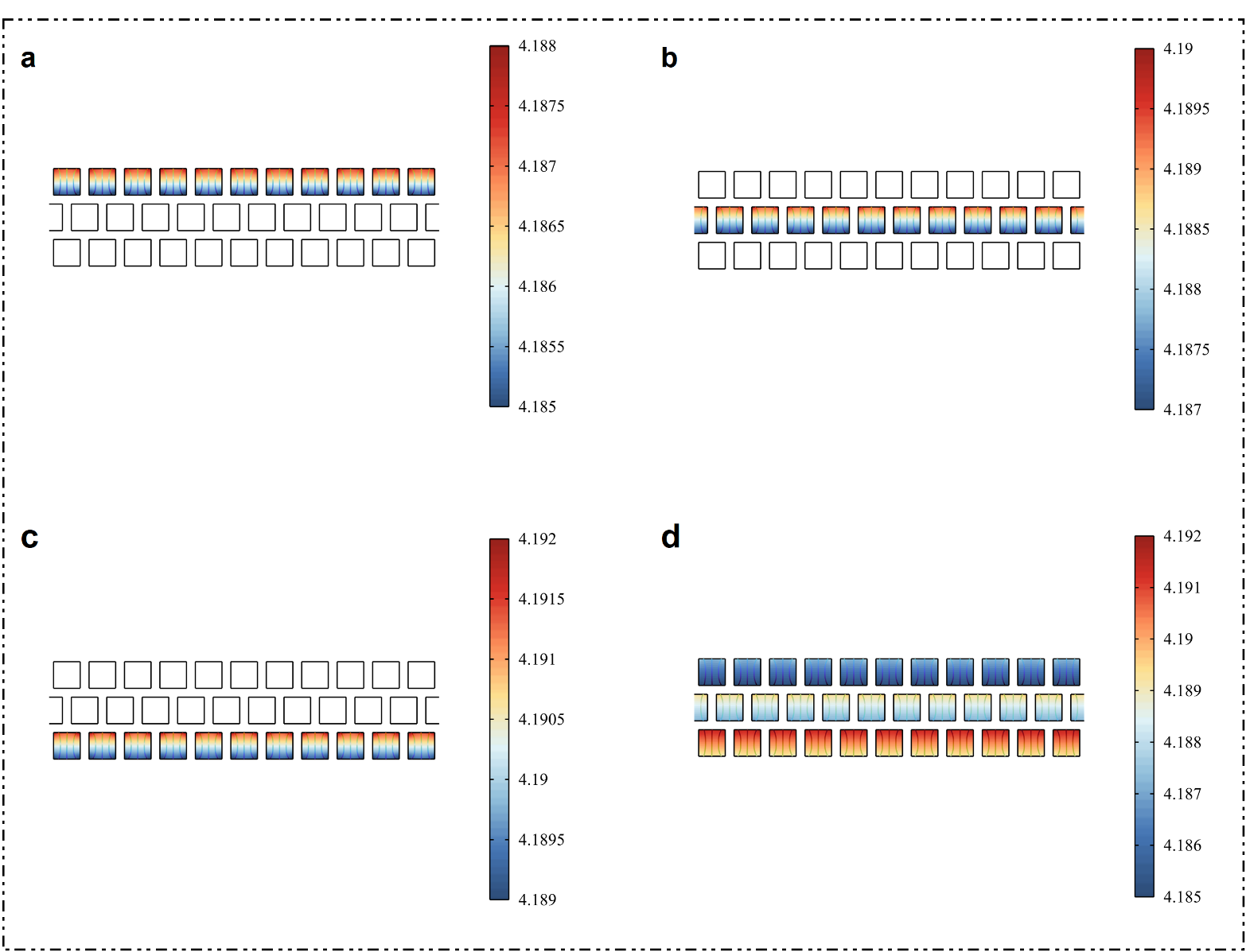


**Figure S17.** Simulation results of the internal electric field of (a-c) individual single sites and (d) the total distribution of BTO particle sites at the PCNCM83/LPSC1.5@BTO interface.


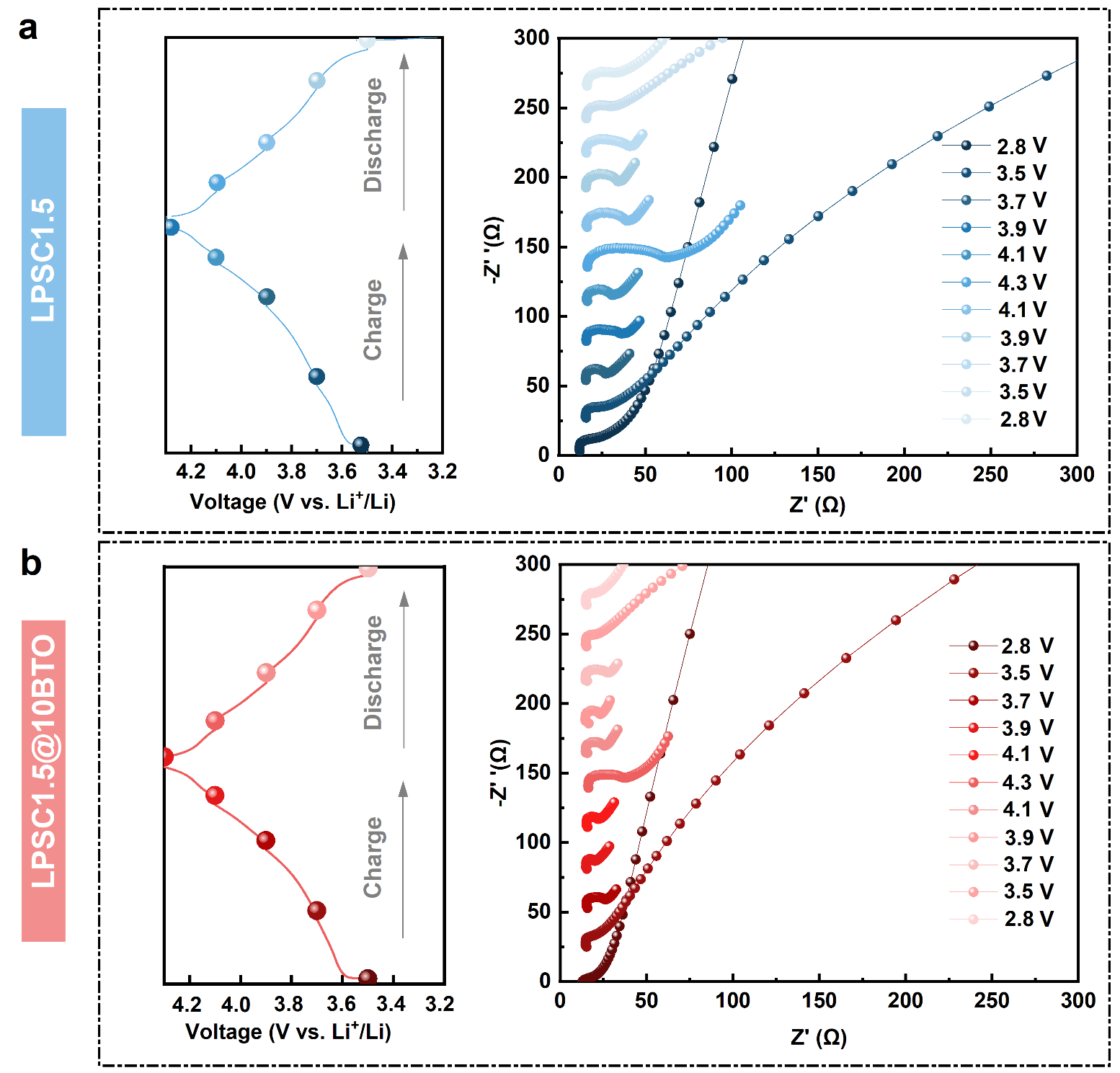


**Figure S18.** In-situ electrochemical impedance spectroscopy (EIS) analysis during the initial charge-discharge process of ASSBs with (a) pristine LPSC1.5 and (b) LPSC1.5@10BTO electrolytes. Galvanostatic charge-discharge profiles (25 °C, 0.1 C, 2.8-4.3 V vs. Li^+^/Li) with key voltage points (2.8 V, 3.5 V, 3.7 V, 3.9 V, 4.1 V, 4.3 V) marked, corresponding to the impedance test timings. Corresponding Nyquist plots at the marked voltage points.


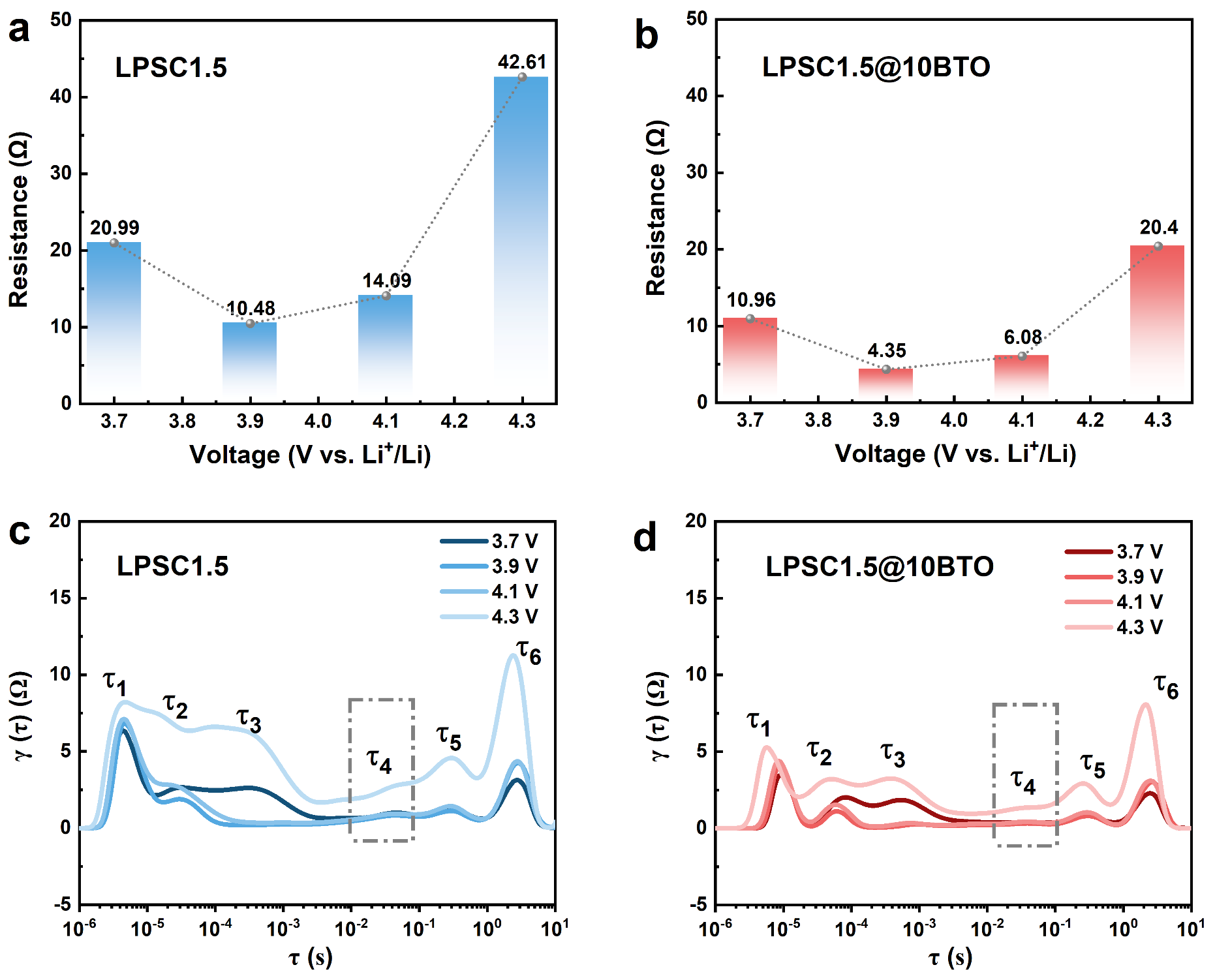


**Figure S19.** Voltage-dependent impedance characteristics of ASSBs with different electrolytes. (a) Voltage-dependent impedance of the LPSC1.5-based cell and (b) that of the LPSC1.5@10BTO-based cell; tests were conducted at 25 °C within the voltage range of 2.8-4.3 V vs. Li^+^/Li, in which the measured resistance values correspond to the total impedance of the respective cells. Distribution of Relaxation Times (DRT) profiles derived from EIS measurements for (c) LPSC1.5-based ASSBs and (d) LPSC1.5@10BTO-based ASSBs at different charging voltages (3.7 V, 3.9 V, 4.1 V, 4.3 V vs. Li^+^/Li).


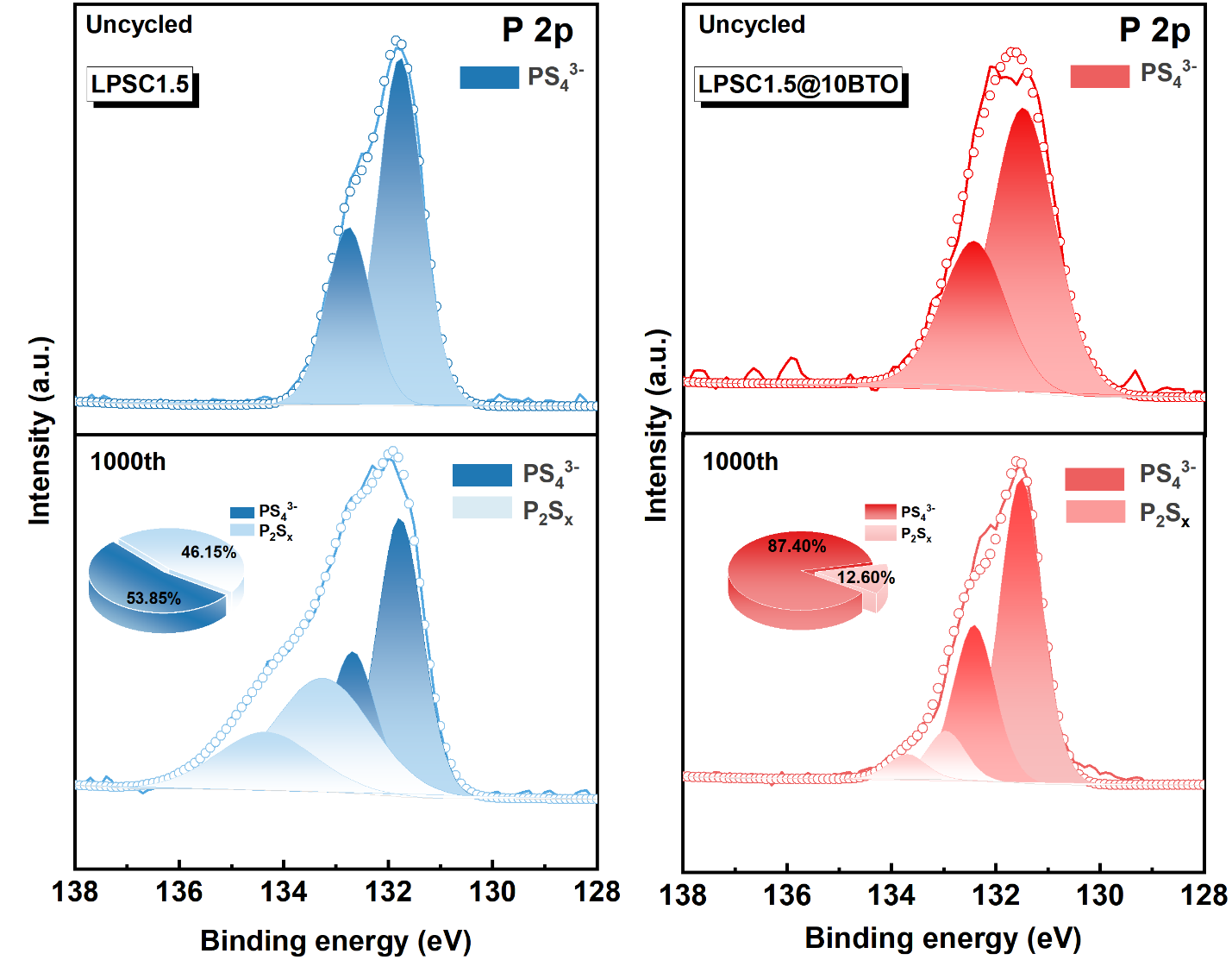


**Figure S20.** XPS characterization of P 2p for (a) PCNCM83/LPSC1.5 and (b) PCNCM83/LPSC1.5@10BTO composite after 1000 cycles at 1 C.


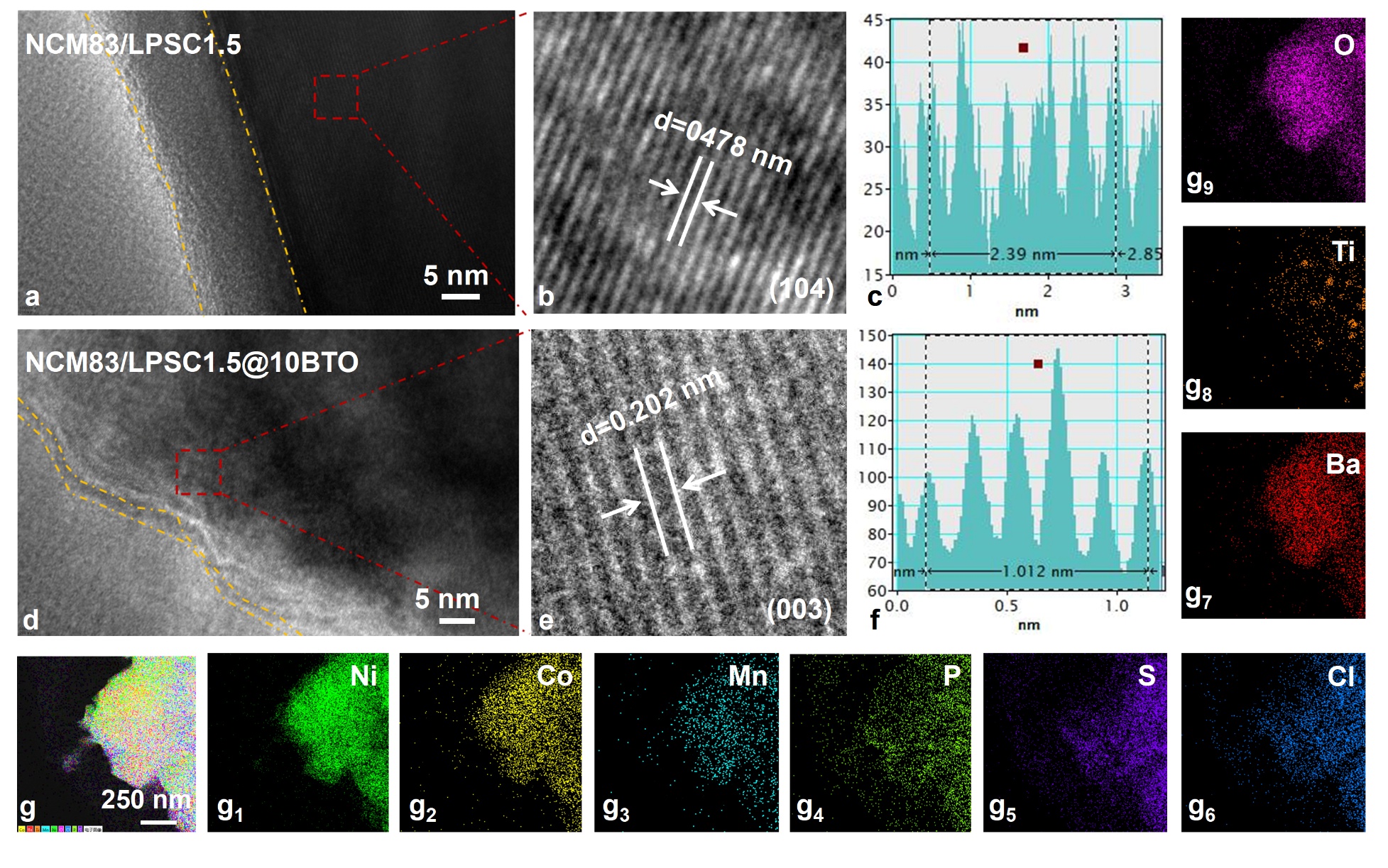


**Figure S21.** TEM images and the corresponding interplanar spacings of (a-c) PCNCM83/LPSC1.5 and (d-f) PCNCM83/LPSC1.5@10BTO composite after 1000 cycles at 1 C with (g) EDS mappings of Ni, Co, Mn, Ti, Ba, O, P, S, Cl of PCNCM83/LPSC1.5@10BTO composite.


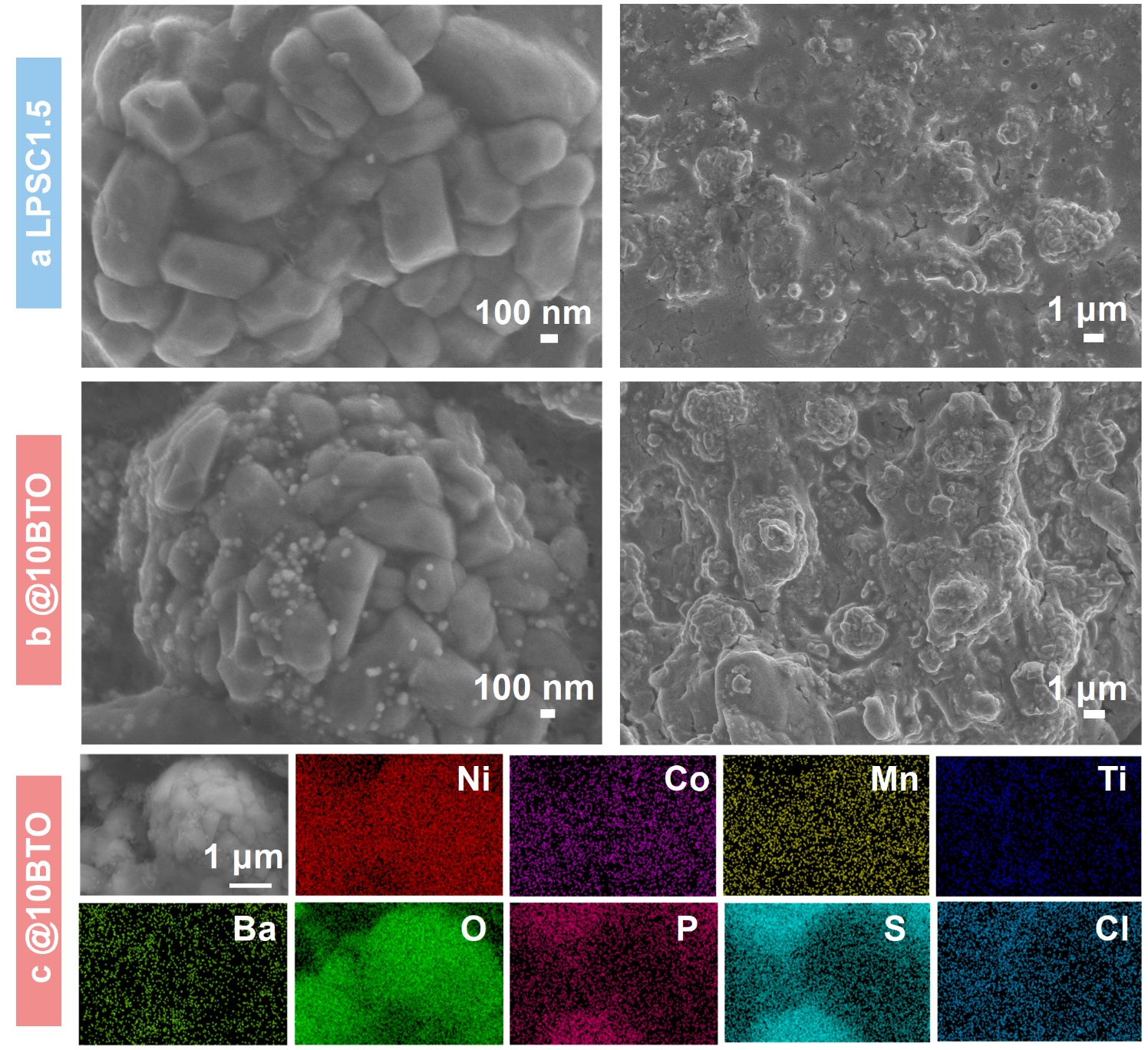


**Figure S22.** SEM images of PCNCM83/LPSC1.5 and PCNCM83/LPSC1.5@10BTO composite after 1000 cycles at 1 C with EDS mappings of Ni, Co, Mn, Ti, Ba, O, P, S, Cl of PCNCM83/LPSC1.5@10BTO composite.


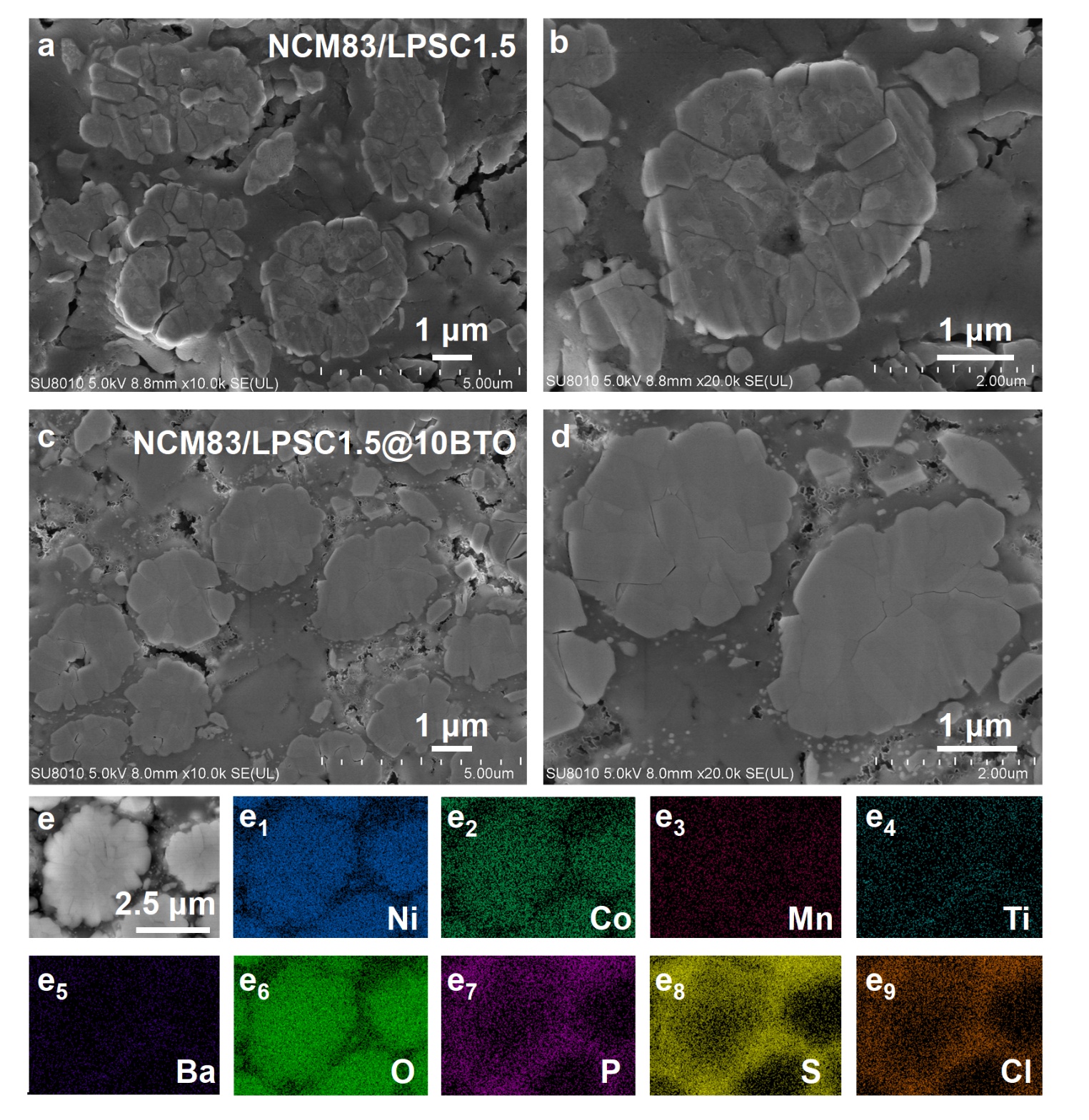


**Figure S23.** Cross-sectional images of (a, b) PCNCM83/LPSC1.5 and (c, d) PCNCM83/LPSC1.5@10BTO composite after 1000 cycles at 1 C with (e) EDS mappings of Ni, Co, Mn, Ti, Ba, O, P, S, Cl of PCNCM83/LPSC1.5@10BTO composite.


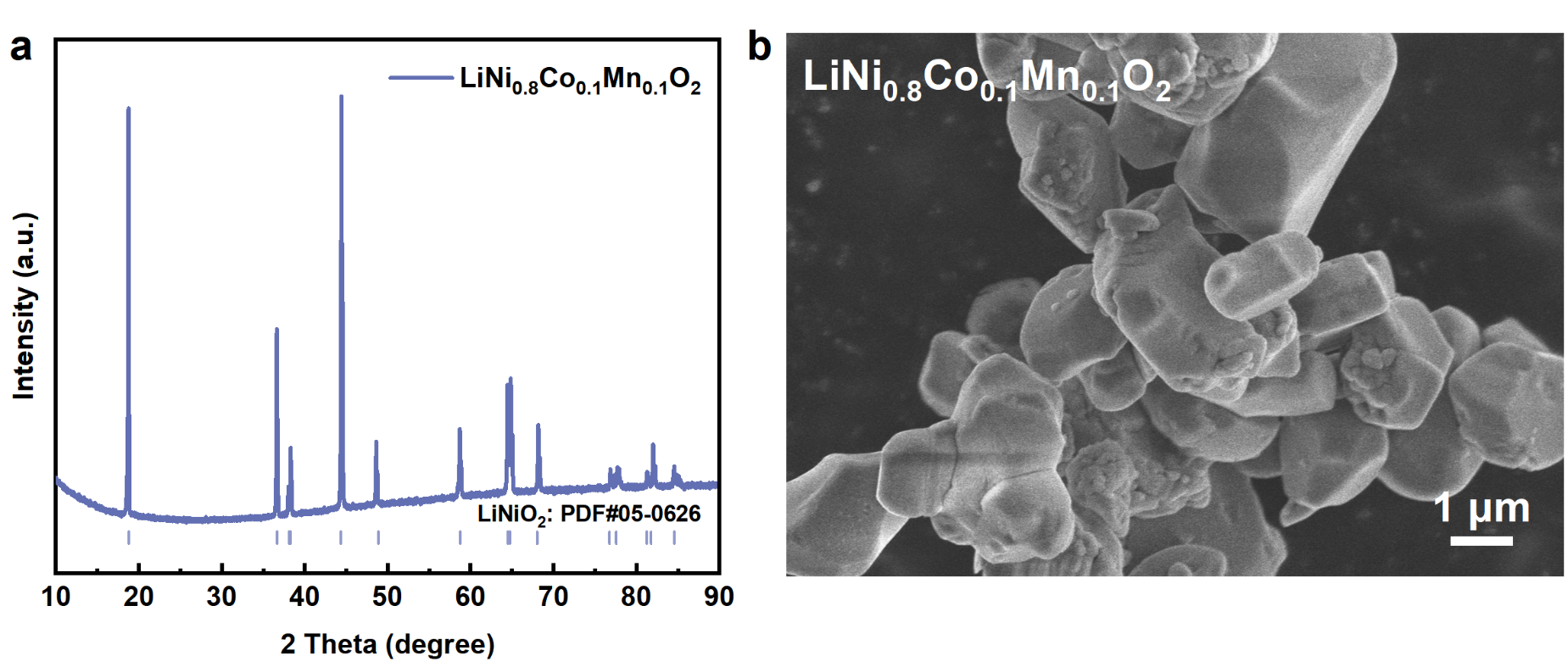


**Figure S24.** (a) XRD pattern and (b) SEM image of commercial SCNCM811.


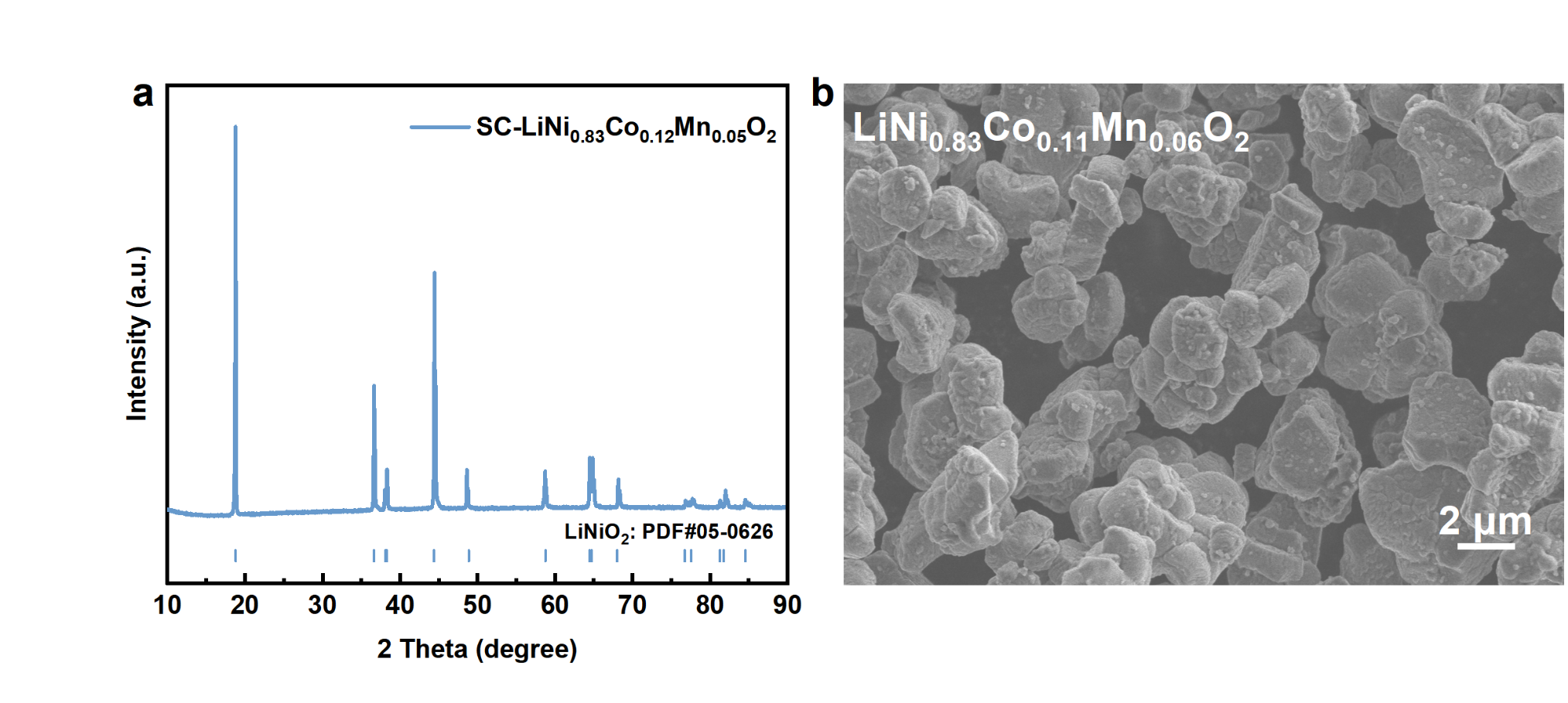


**Figure S25.** (a) XRD pattern and (b) SEM image of commercial SCNCM83.


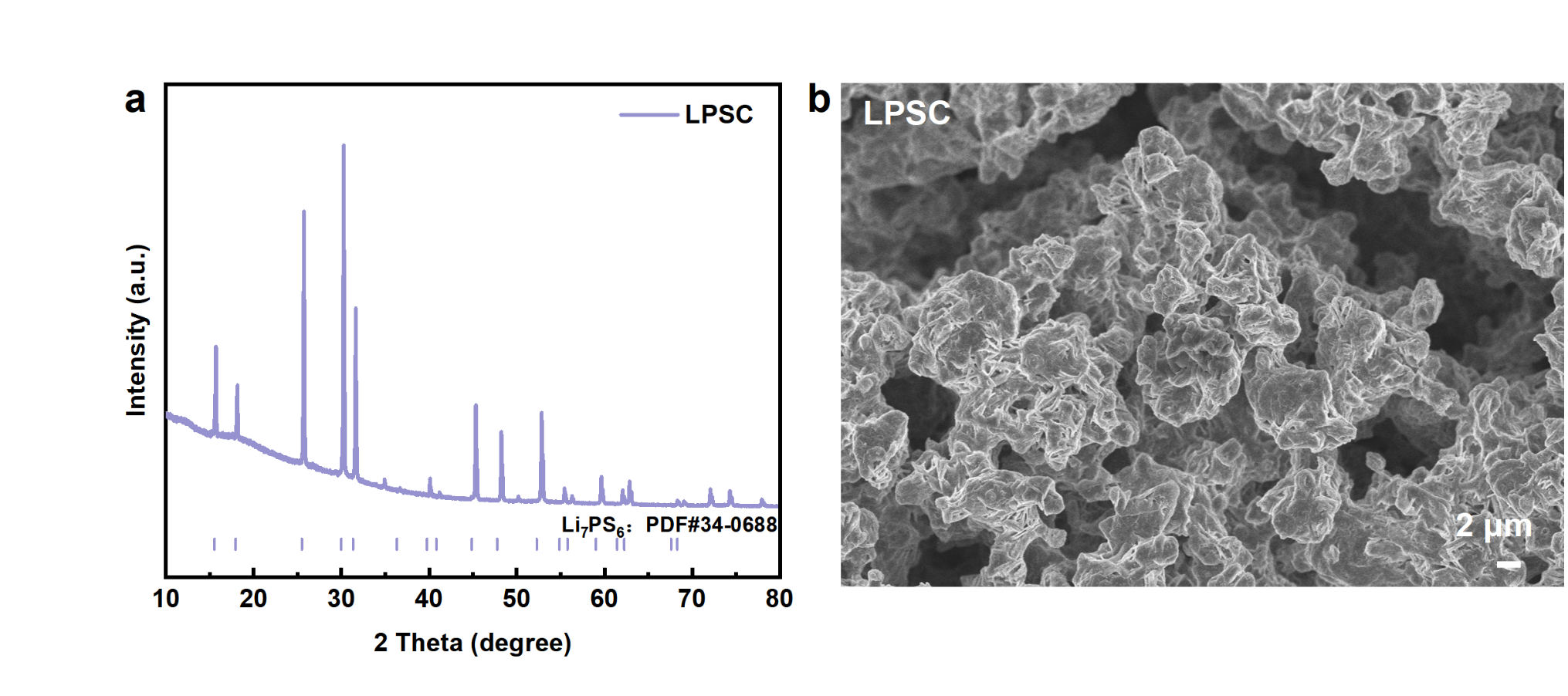


**Figure S26.** (a) XRD pattern and (b) SEM image of commercial LPSC.

**
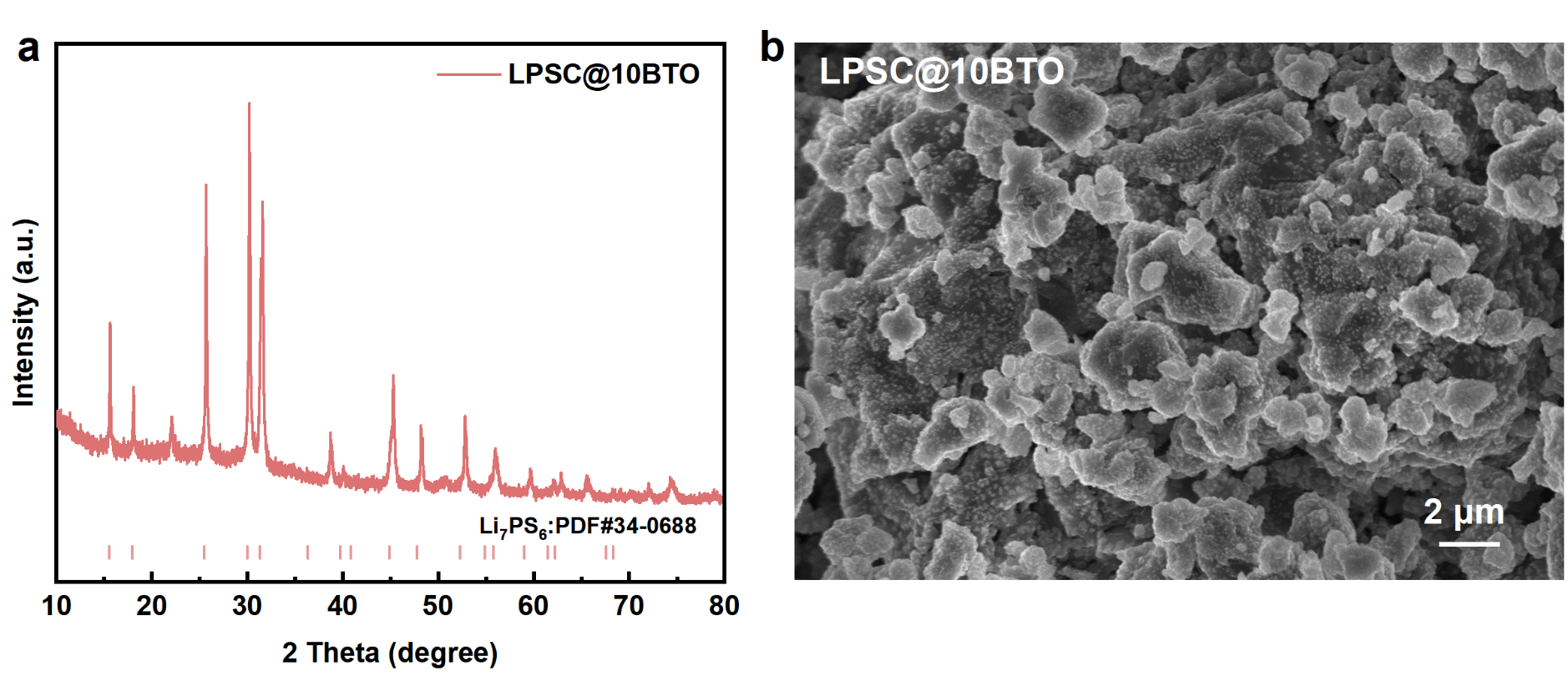
**

**Figure S27.** (a) XRD pattern and (b) SEM image of LPSC@10BTO.


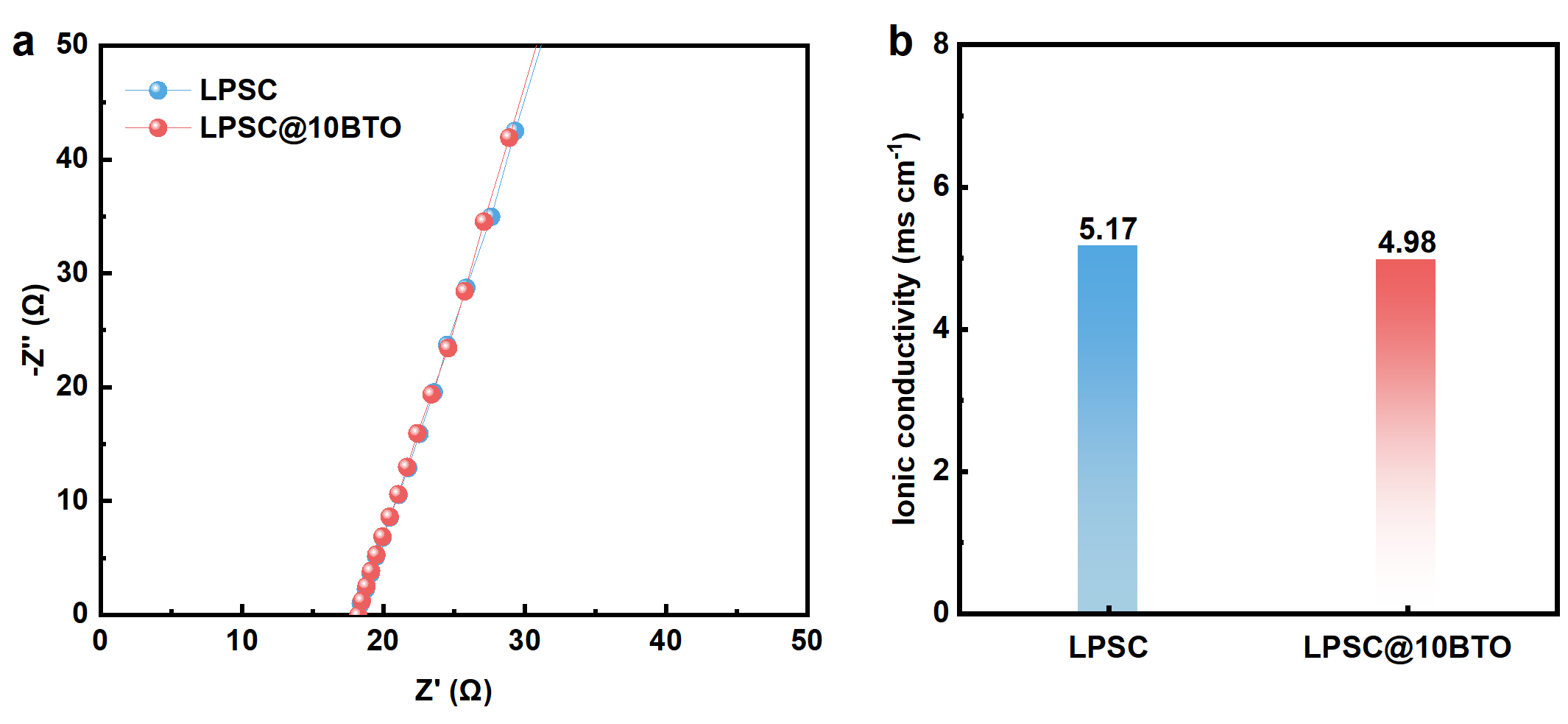


**Figure S28.** (a) The Nyquist plots and (b) calculated ionic conductivity of LPSC1.5 and LPSC1.5@10BTO.

**
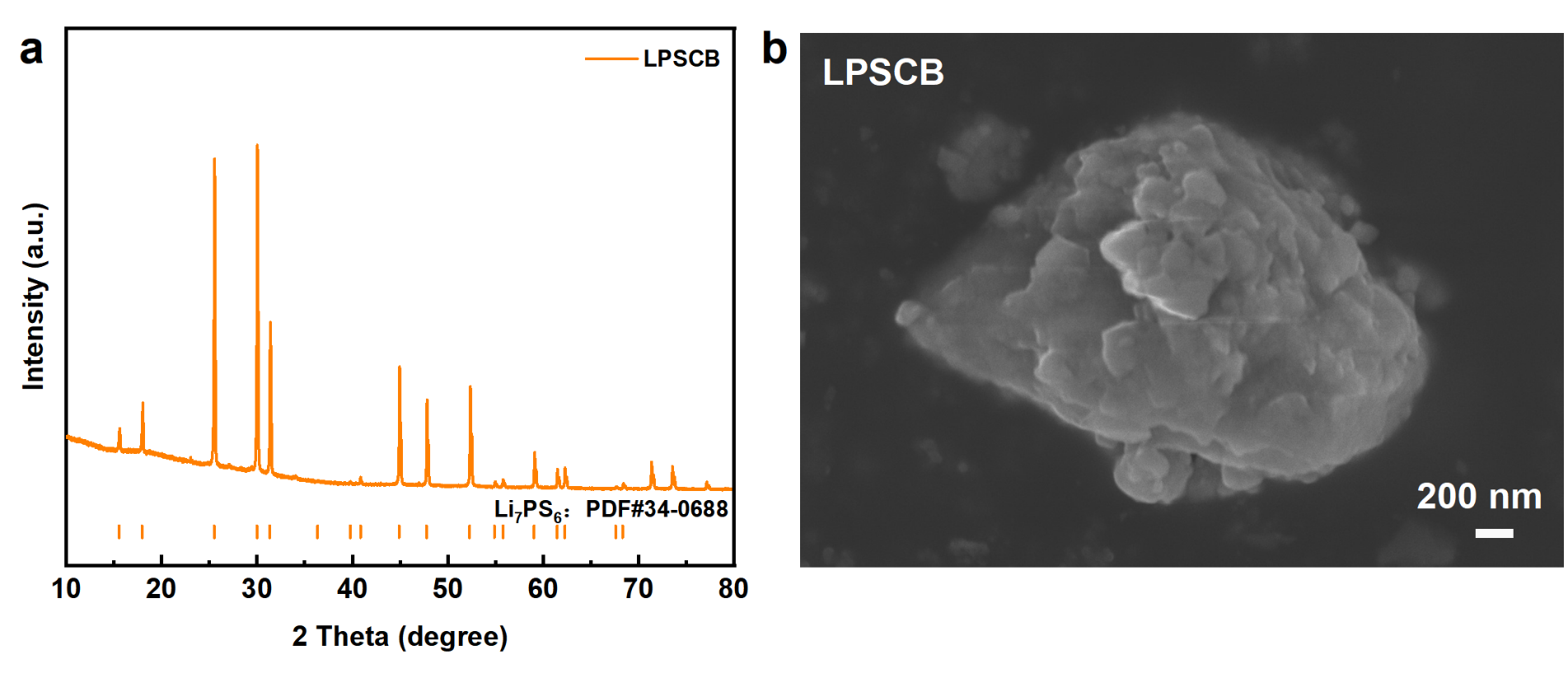
**

**Figure S29.** (a) XRD pattern and (b) SEM image of commercial LPSCB.

**
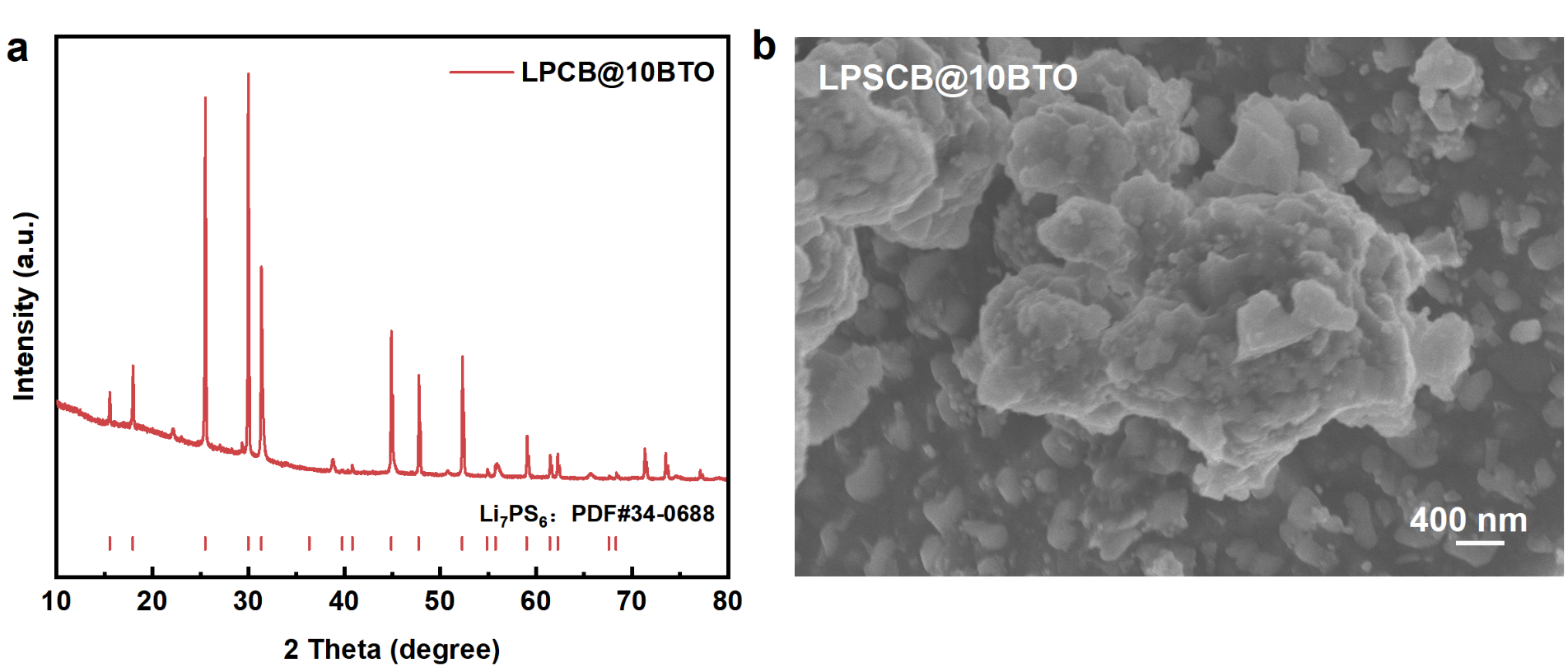
**

**Figure S30.** (a) XRD pattern and (b) SEM image of LPSCB@10BTO.


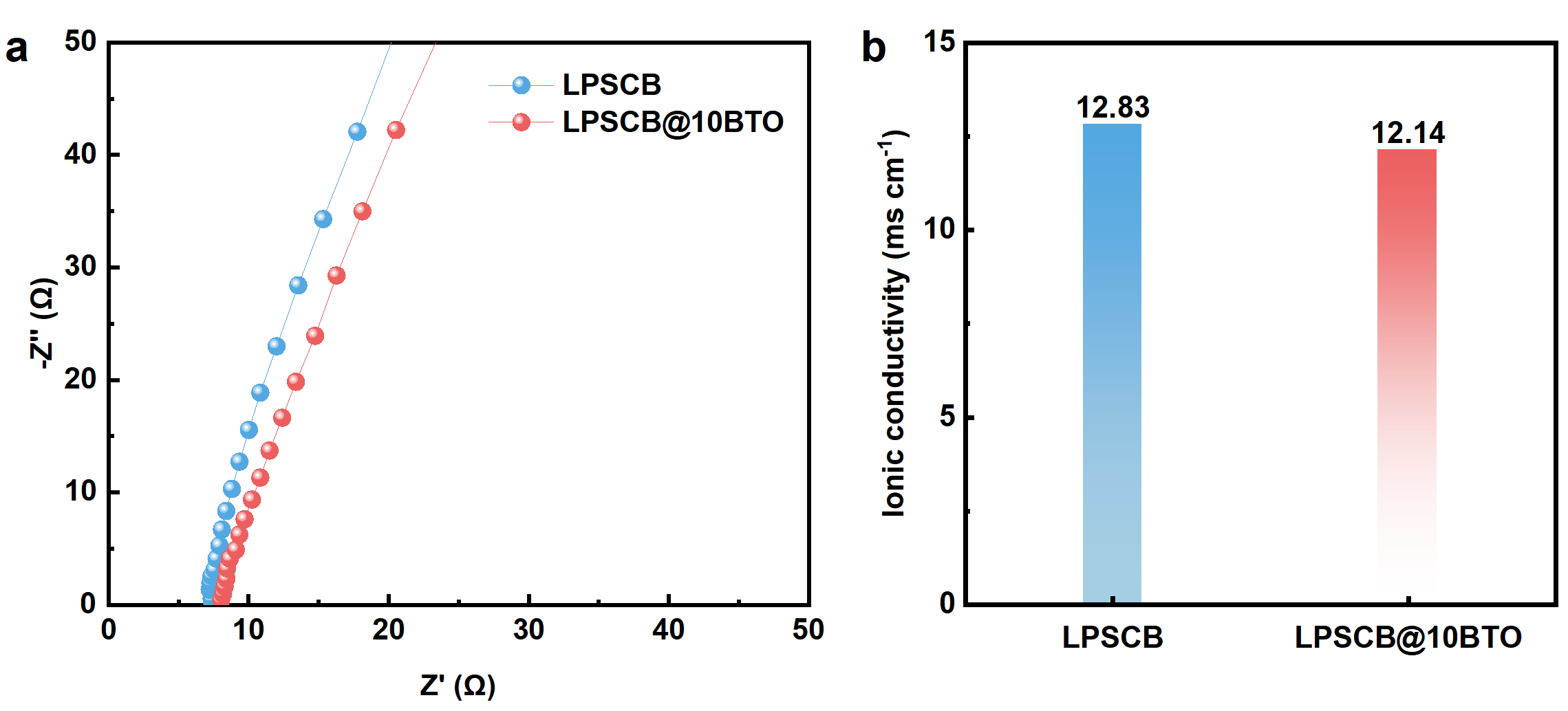


**Figure S31.** (a) The Nyquist plots and (b) calculated ionic conductivity of LPSCB and LPSCB @10BTO.


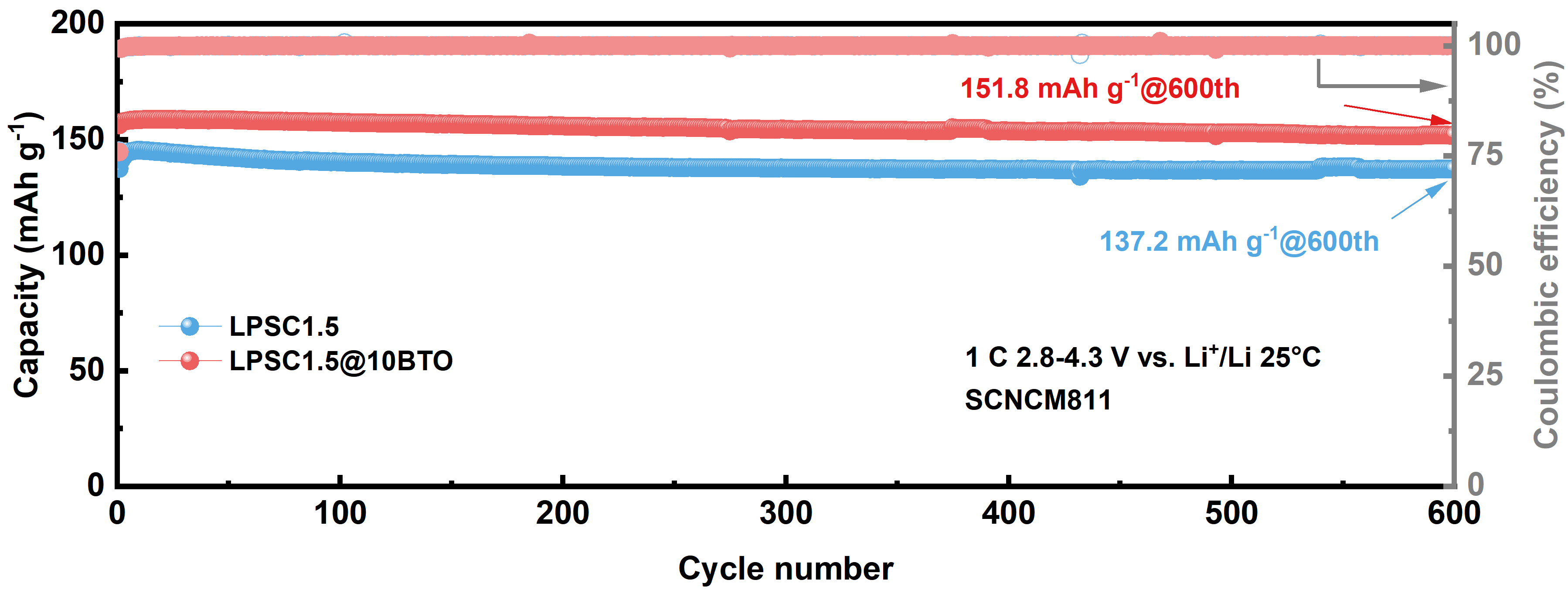


**Figure S32.** Cycle performance of SCNCM811/LPSC1.5@xBTO (x = 0, and 10)/LiIn at 1 C within the voltage range of 2.8-4.3 V vs. Li^+^/Li.

**
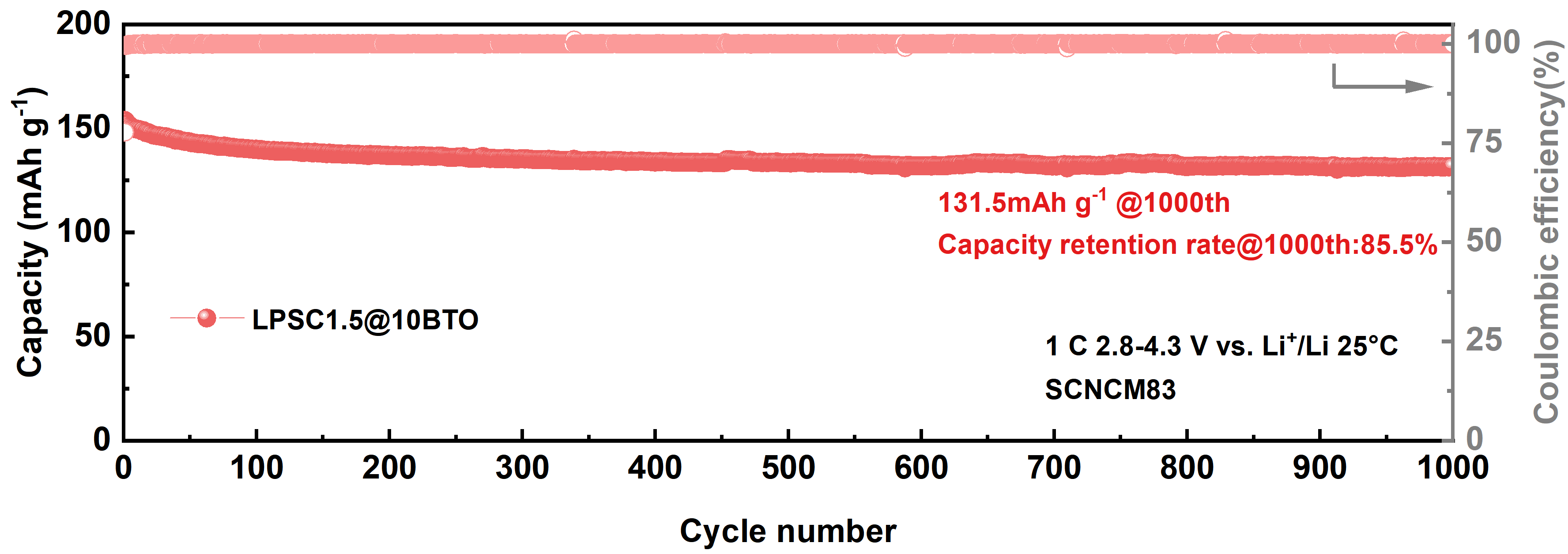
**

**Figure S33.** Cycle performance of SCNCM83/LPSC1.5@10BTO/LiIn at 1 C within the voltage range of 2.8-4.3 V vs. Li^+^/Li.


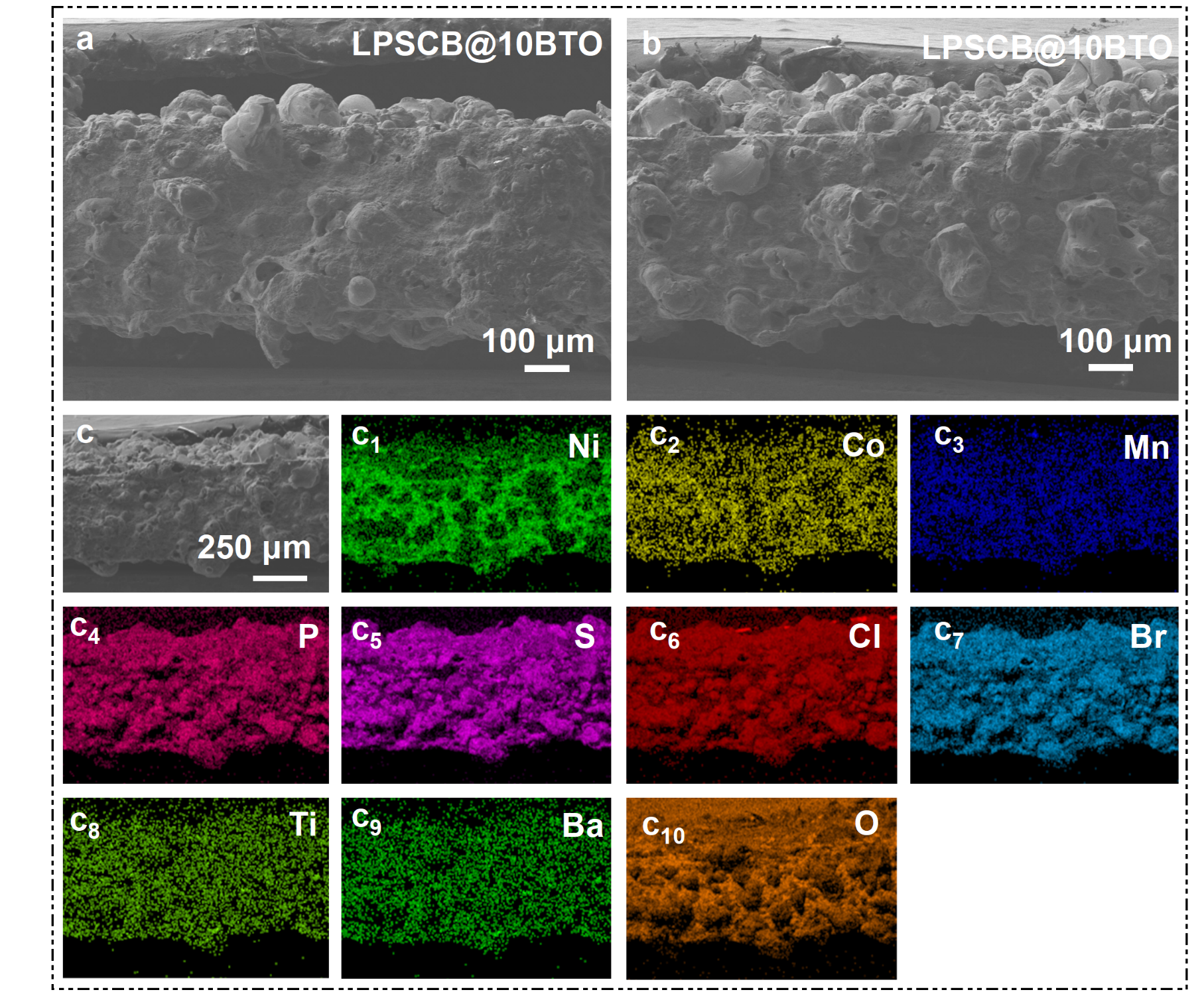


**Figure S34.** Cross-sectional SEM image and corresponding mappings of cycled PCNCM83/LPSCB@10BTO with a cathode active material mass loading of 56.27 mg cm^-2^.


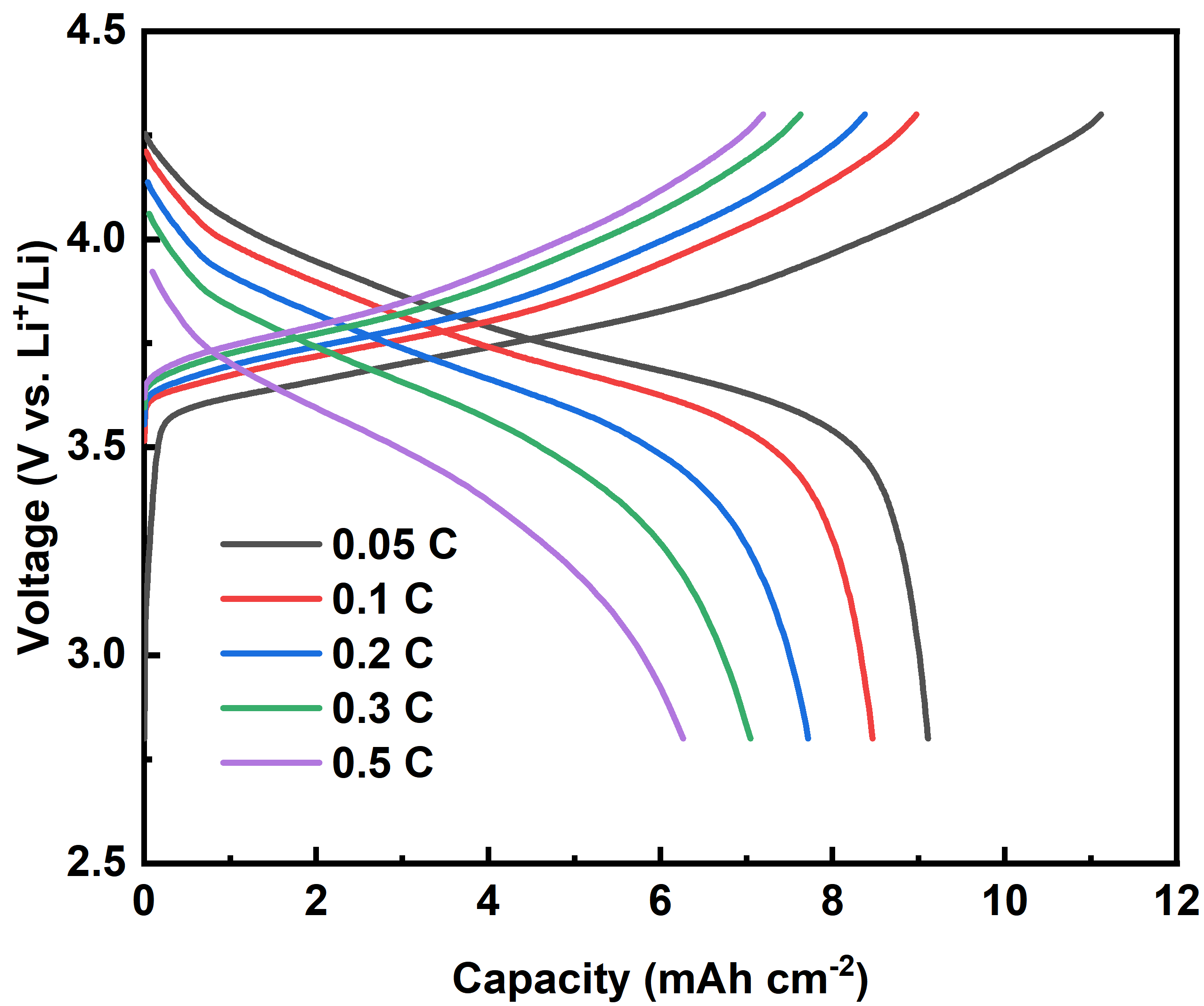


**Figure S35.** Charge-discharge profiles of PCNCM83/LPSCB@10BTO/LiIn with a cathode active material mass loading of 56.27 mg cm^-2^.

2. Supplementary Tables

**Table S1.** The raw material costs (ton-level prices) of different types of lithium-stable sulfide electrolytes and nano BaTiO_3_. The prices are taken from commercial suppliers [1, 2].

| **Chemical name and description** | **Unit price, [$/kg]** |
| --- | --- |
| Li_2_S, 99% | 662.37 |
| P_2_S_5_, 99.5% | 22.10 |
| LiCl, 99% | 13.79 |
| LiBr, 99% | 27.96 |
| BaTiO_3_, 99.9% | ~20 |

**Table S2.** Raw materials costs of different sulfide solid electrolytes were estimated using the bulk prices listed in Table S1.

| **Solid electrolyte** | **Raw materials cost, [$/kg]** | **Cost reduction (%)** |
| --- | --- | --- |
| Li_6_PS_5_Cl | 212 | 0 |
| Li_6_PS_5_Cl@5BTO | 202.9 | 4.3 |
| Li_6_PS_5_Cl@10BTO | 194.6 | 8.2 |
| Li_6_PS_5_Cl@15BTO | 187 | 11.8 |
| Li_5.5_PS_4.5_Cl_1.5_ | 189 | 0 |
| Li_5.5_PS_4.5_Cl_1.5_@5BTO | 181 | 4.2 |
| Li_5.5_PS_4.5_Cl_1.5_@10BTO | 173.6 | 8.1 |
| Li_5.5_PS_4.5_Cl_1.5_@15BTO | 167 | 11.6 |
| Li_5.5_PS_4.5_Cl_0.8_Br_0.7_ | 190 | 0 |
| Li_5.5_PS_4.5_Cl_0.8_Br_0.7_@5BTO | 181.9 | 4.3 |
| Li_5.5_PS_4.5_Cl_0.8_Br_0.7_@10BTO | 174.6 | 8.1 |
| Li_5.5_PS_4.5_Cl_0.8_Br_0.7_@15BTO | 167.8 | 11.7 |

| **Table S3.** Summary of reported rate performances of argyrodite-based full cells. | | | | | | | | |
| --- | --- | --- | --- | --- | --- | --- | --- | --- |
| **Ref.** | **Cathode materials** | **Loading mass of active material (mg cm^-2^)** | **Solid-state electrolytes** | **Discharge capacity (mAh g^-1^)** | | | | |
|  |  |  |  | **0.1 C** | **1 C** | **3 C** | **5 C** | **10 C** |
| **Our work** | **PCNCM83** | **6** | **LPSC1.5@10BTO** | **169.7** | **146.2** | **129.2** | **114.8** | **81.7** |
| [3] | SCNCM622 | 2.5 | LPSC1.5 | 179.4 | 127.8 | 110.5(2C) | 80.8 | 51.1 |
| [4] | SCNCM83@Li_3_BO_3_ | 7.64 | LPSCB | 167 | 129 | 103(2C) | - | - |
| [5] | SCNCM811 | 6.4 | LPSC1.5 | 183.2 | 131.6 | 108.5(2C) | - | - |
| [6] | SCNCM811@ 0.5Li_2_O·0.5B_2_O_3_ | 10 | LPSC | 209 | 152 | 74(2C) | - | - |
| [7] | PCNCM712@LiNbO_3_ | 4.46 | Li_6_P_0.85_Si_0.05_Ge_0.05_Sn_0.05_S_4.5_BrCl_0.5_ | 184.2 | 157.5 | 134.2 | 116.4 | 67.7 |
| [8] | SCNCM9055 | 6.2 | LPSC | 195.5 | 153.9 | 100 | 43.9 | - |
| [9] | PCNCM811@Li_7_TaO_6_ | 8.92 | LPSC | 203.02 | 125 | 55 | - | - |
| [10] | PC-  NCM811@Li_2_O | 8.92 | LPSC | 170.81 | 110 | 60 | - | - |

**Table S4.** Summary of reported cycling performances of argyrodite-based full cells.

| **Ref.** | **Cathode materials** | **Solid-state electrolytes** | **Loading mass of active material (mg cm^-2^)** | **Current density (mA cm^-2^)** | **Capacity**  **(mAh g^-1^)** | **Cycle numbers** | **Capacity retention (%)** |
| --- | --- | --- | --- | --- | --- | --- | --- |
| **Our work** | **PCNCM83** | **LPSC1.5@10BTO** | **5.79** | **1 C** | **150.5(1st)** | **1000** | **94.9** |
|  |  |  | **5.62** | **3 C** | **121.4(1st)** | **5000** | **85.1** |
|  |  |  | **5.79** | **5 C** | **96.8(1st)** | **10000** | **83.5** |
|  |  |  | **5.80** | **7 C** | **42.1(1st)** | **10000** | **151.3** |
|  |  |  | **5.79** | **10 C** | **36.3(1st)** | **3000** | **148.5** |
|  | **SCNCM811** | **LPSC1.5@10BTO** | **5.44** | **3 C** | **133.6(1st)** | **2500** | **96.1** |
|  | **PCNCM83** | **LPSC@10BTO** | **5.08** | **3 C** | **102.6(1st)** | **2026** | **88.1** |
|  | **PCNCM83** | **LPSCB@10BTO** | **5.71** | **5 C** | **110.6(1st)** | **4000** | **87.3** |
|  | **SCNCM83** | **LPSC1.5@10BTO** | **5.79** | **1 C** | **153.8(1st)** | **1000** | **85.5** |
| [3] | SCNCM622 | LPSC1.5 | 2.5 | 10 C | 66.6(1st) | 4500 | 82.4 |
| [4] | SCNCM83@Li_3_BO_3_ | LPSC1.5 | 7.13 | 1 C | 116(101th) | 7500 | 96 |
|  |  | LPSCB | 7.64 | 2 C | 122 | 1800 | 100 |
| [5] | SCNCM811 | LPSC1.5 | 2.5 | 5 C | 120.2 | 3500 | 64 |
| [6] | SCNCM811@ 0.5Li_2_O·0.5B_2_O_3_ | LPSC | 10 | 1 C | 155 | 1000 | 87.8 |
| [7] | PC- NCM712@LiNbO_3_ | Li_6_P_0.85_Si_0.05_Ge_0.05_Sn_0.05_S_4.5_BrCl_0.5_ | 4.46 | 1 C | 121.4(1st) | 450 | 80 |
|  |  |  |  | 3 C | 111.1(1st) | 900 | 79.2 |
|  |  |  |  | 5 C | 96.1(1st) | 1400 | 85.6 |
| [8] | SCNCM9055 | LPSC | 6.2 | 3 C | 120.32 | 2400 | 81.67 |
| [9] | PC-  NCM811@Li_7_TaO_6_ | LPSC | 8.92 | 1 C | 131.25(41th) | 5650 | 61.1 |
| [10] | PC-  NCM811@Li_2_O | LPSC | 8.92 | 1 C | 117(36th) | 1290 | 94 |

**References**

[1] H. Li, Q. Lin, J. Wang, L. Hu, F. Chen, Z. Zhang, C. Ma, *Angew. Chem. Int. Ed.* **2024**, *63*, e202407892.

[2] X. Zhang, H. Yi, Y. Shi, K. Lu, M. Zhang, D. Zhang, H. Zeng, S. Zhang, D. Ji, P. Chen, B. Xu, C. Tian, Z. Li, X. Zhang, *Energy Storage Mater.* **2025**, *83*, 104689.

[3] L. Peng, C. Yu, Z. Zhang, H. Ren, J. Zhang, Z. He, M. Yu, L. Zhang, S. Cheng, J. Xie, *Chem. Eng. J.* **2022**, *430*, 132896.

[4] S. Wang, C. Lou, X. Wu, J. Lin, A. Gautam, S. Li, J. Huang, Z. Cheng, S. Zhang, X. Zhang, F. Strauss, T. Brezesinski, G. Luo, M. Tang, Y. Shen, Y. Lin, C.-W. Nan, *Matter* **2025**, *8*, 102135.

[5] L. Peng, S. Chen, C. Yu, C. Liao, M. Sun, H.-L. Wang, L. Zhang, S. Cheng, J. Xie, *J. Power Sources* **2022**, *520*, 230890.

[6] J. Luo, B. Guo, N. Li, Q. Huang, J. Wang, Y. Fu, L. Jia, X. Zhang, J. Hou, J. Zhu, X. Zhuang, *Nat. Commun.* **2025**, *16*, 9462.

[7] W. Li, Z. Chen, Y. Chen, L. Zhang, G. Liu, L. Yao, *Adv. Funct. Mater.* **2024**, *34*, 2312832.

[8] C. Liu, W. Li, K. Deng, P. An, R. Wang, B. Chen, Q. Xiao, J. Wu, L. Yao, G. G. Liu, *Chem. Eng. J.* **2025**, *517*, 164576.

[9] J. Shi, Z. Ma, K. Han, Q. Wan, D. Wu, X. Qu, P. Li, *J. Mater. Chem. A* **2022**, *10*, 21336-21348.

[10] J. Shi, P. Li, K. Han, D. Sun, W. Zhao, Z. Liu, G. Liang, K. Davey, Z. Guo, X. Qu, *Energy Storage Mater.* **2022**, *51*, 306-316.
